# Frequent Recombination Events in *Leishmania donovani*: Mining Population Data

**DOI:** 10.3390/pathogens9070572

**Published:** 2020-07-15

**Authors:** Igor B. Rogozin, Arzuv Charyyeva, Ivan A. Sidorenko, Vladimir N. Babenko, Vyacheslav Yurchenko

**Affiliations:** 1 National Center for Biotechnology Information, National Library of Medicine, National Institutes of Health, Bethesda, MD 20894, USA; rogozin@ncbi.nlm.nih.gov; 2 Life Science Research Centre, Faculty of Science, University of Ostrava, 710 00 Ostrava, Czech Republic; arzuvc@gmail.com; 3 Institute of Cytology and Genetics, 630090 Novosibirsk, Russia; vanyasidorenko22@gmail.com (I.A.S.); bob@bionet.nsc.ru (V.N.B.); 4 Martsinovsky Institute of Medical Parasitology, Tropical and Vector Borne Diseases, Sechenov University, 119435 Moscow, Russia

**Keywords:** gene conversion, *Leishmania donovani* species complex, whole-genome sequencing, concerted evolution

## Abstract

The *Leishmania donovani* species complex consists of all *L. donovani* and *L. infantum* strains mainly responsible for visceral leishmaniasis (VL). It was suggested that genome rearrangements in *Leishmania* spp. occur very often, thus enabling parasites to adapt to the different environmental conditions. Some of these rearrangements may be directly linked to the virulence or explain the reduced efficacy of antimonial drugs in some isolates. In the current study, we focused on a large-scale analysis of putative gene conversion events using publicly available datasets. Previous population study of *L. donovani* suggested that population variability of *L. donovani* is relatively low, however the authors used masking procedures and strict read selection criteria. We decided to re-analyze DNA-seq data without masking sequences, because we were interested in the most dynamic fraction of the genome. The majority of samples have an excess of putative gene conversion/recombination events in the noncoding regions, however we found an overall excess of putative intrachromosomal gene conversion/recombination in the protein coding genes, compared to putative interchromosomal gene conversion/recombination events.

## 1. Introduction

The *Leishmania donovani* species complex consists of all *L. donovani* and *L. infantum* strains responsible for visceral leishmaniasis (VL) [1,2]. Besides VL, atypical cutaneous manifestations caused by both species of the complex have been also reported [3,4]. While *L. donovani* is considered to be an anthroponotic agent, *L. infantum* is zoonotic, with dogs and numerous wild mammals being involved in the disease transmission [5]. Both species are widespread with known major foci for *L. donovani* and *L. infantum* being the India/East Africa and the Middle East, respectively [6]. Although *L. chagasi* in the New World was historically referred to as a separate species, recent studies have demonstrated that it is a mere subpopulation of *L. infantum* [7], which had been probably spread in the Americas via migration in the 15th–16th century [8]. The clinical manifestations of the leishmaniasis caused by *L. donovani* spp. complex differ depending on the immune status of the infected individuals, parasite strains and transmitting sand fly vector’s species [4,9].

It was suggested that genome rearrangements in *Leishmania* spp. occur very often, thus enabling parasites to adapt to the different environmental conditions [10,11]. Some of these rearrangements may be directly linked to the virulence [12,13] or explain the reduced efficacy of antimonial drugs in some isolates [14]. Below, we will discuss some prominent examples attributed to genome rearrangements in trypanosomatids, with a particular focus on *Leishmania* spp. 

Lipophosphoglycan (LPG) is one of the most abundant surface glycoconjugates, which is mainly involved in parasite colonization of the vector’s midgut [15]. The LPG molecules are differentially modified during the development, facilitating proper parasites’ migration, evasion of the host immune system, and promoting the host specificity [16,17]. Tandem arrangement of the genes, encoding LPG modifying enzymes, provides a strong evidence of gene conversion in *Leishmania* spp. [18,19]. Of note, this model of organization is also conserved in monoxenous (=one host [20,21]) relatives of *Leishmania* of the subfamily Leishmaniinae [22]. 

Variant surface glycoproteins (VSGs) facilitate immune evasion, while VSG-encoding genes define antigenic variation in trypanosomes [23]. These genes have likely evolved as a result of several gene conversion events [24,25].

One of the essential virulence factors, the glycoprotein 63 (GP63), is encoded in a variable number of copies in different species of *Leishmania* [26]. All *Leishmania* spp., sequenced thus far, harbor at least two long, as well as variable numbers of short GP63-encoding sequences. This suggests that mosaic gene conversion has a high impact on the evolutionary history of these species [26,27]. Other prominent examples of this process in *Leishmania* are genes encoding cysteine proteases [28], *hsp70* gene cluster [29], amastins and A2-A2rel gene clusters [30,31]. 

In the current study we focused on a large-scale analysis of putative gene conversion events using publicly available datasets. Previous population study of *L. donovani* suggested that population variability of *L. donovani* is fairly low, however the authors used masking procedures and strict read selection criteria [14,32]. We decided to reanalyze DNA-seq data without masking sequences, because we were interested in the most dynamic fraction of the genome. A substantial variability of some regions of the *L. donovani* genome was documented. The majority of samples have an excess of putative gene conversion events in the noncoding regions, however we found an overall excess of putative intrachromosomal gene conversion/recombination in the protein coding genes, compared to putative interchromosomal gene conversion/recombination events.

## 2. Results

### 2.1. Analysis of Leishmania donovani DNA-seq Data

**Figure 1:**
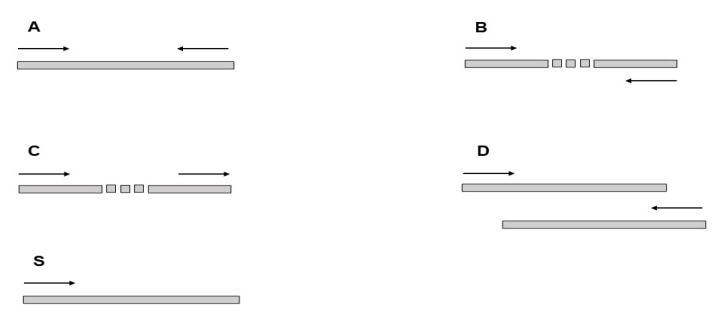
Schematic representation of **A**—(paired end reads), **B**—(putative intrachromosomal gene conversion events), **C**—(inversions), **D**—(putative interchromosomal gene conversion events), and **S**—(sole mapped) type reads.

We studied five types of read configurations (Figure 1) in 28 *Leishmania donovani* genomes. The number of B-type reads (putative intra-chromosomal gene conversion events, see Materials and Methods for definitions) is approximately equal to that of the S-type reads (sole mapped reads) and substantially greater than that for the C- and D-type reads (inversion and putative inter-chromosomal gene conversion events, respectively) (Table 1 and Table 2). The fraction of B-type and S-type reads that overlap with protein-coding genes is also similar and significantly larger than the corresponding values for the C- and D-type reads. 

To test the possibility that the reason for the observed B-type reads prevalence are because of errors of read mapping with numerous mismatches (for example, low-quality sequencing), we have analyzed reads with the similarity level 90–95%. We found only a few reads that were largely located in noncoding DNA (Table 3). Thus, it is unlikely that low-quality reads make a substantial contribution to the B-type reads located in protein-coding genes.

### 2.2. Gradient of Putative Recombination Events Across Protein-Coding Genes

**Figure 2:**
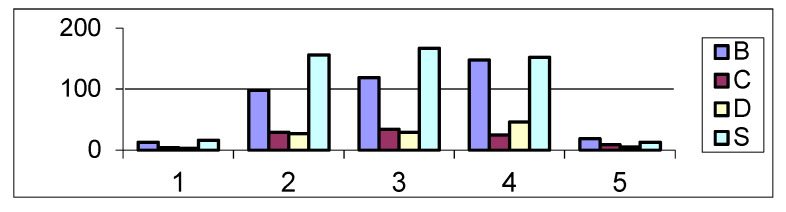
Distribution of B-, C-, D- and S-type reads across protein-coding genes. Number of reads is shown on the Y axis. Bins 1 and 5 correspond to 500 bases of 5′ and 3′ noncoding regions. Protein-coding regions were split into three 33% terciles. Bins 2, 3 and 4 correspond to the first, second and third terciles. We allowed overlaps with protein coding regions for bins 1 and 5 (5′ and 3′ flanking regions), these bins were not used for statistical analyses. All reads that overlap with more than one bin were removed from bins 2, 3 and 4.

Polarity is one of the properties of gene conversion: in almost all cases the frequency of conversion exhibits a gradient “across” the gene [33,34,35]. Here, we studied a distribution of reads across protein-coding genes and 500 bases of flanking regions (Figure 2). The frequency of reads (B, C, D, and S) in the 3′ and 5′ UTRs was substantially lower, compared to the coding regions. We observed a polarity of B- and D-type reads with an increased number of reads toward 3′ end of the protein-coding sequences. C- and S-type reads are distributed more uniformly (Figure 2). We compared the distribution of reads in protein coding regions (bins 2, 3 and 4), and the difference between the distributions of B- and S-type reads is statistically significant (P_χ2_ = 0.029, a modified χ^2^ test, see Materials and Methods). No other significant pairwise differences were detected. We also compared the distribution of reads in protein coding regions (bins 2, 3 and 4) to the uniform distribution using the Pearson’s χ^2^ test, the B-type read distribution was significantly different from the uniform distribution (P_χ2_ = 0.006) whereas C-, D- and S-type read distributions were not significantly different from the uniform distribution.

### 2.3. Analyis of B-Type Reads and Putative Adenylate Cyclase Proteins

We extracted B-type reads that overlap with protein-coding genes and do not overlap with S/C/D-type reads. A total of 137 protein-coding genes that overlap with such B-type reads were identified (Supplementary file Table S2). A total of 67 protein-coding genes overlap with two or more B-type reads (Supplementary file Table S2). The gene encoding the XP_003861613.1 protein (conserved in many Kinetoplastida genomes) has the highest number of overlaps with B-type reads (16 reads, Supplementary file Table S2). This gene does not have paralogous sequences in the *Leishmania donovani* genome and it varies across different *L. donovani* strains (~1–2% divergence at the protein level and 18 amino acid deletion/insertion in the first half, Supplementary file Figure S1). The detected B-type reads are likely to represent recombination events across analyzed strains. These B-type reads are located in the 3′ flanking region and at the very end of the coding region (Supplementary file Table S2). The presence of structural variations in the 3′ region of the protein-coding gene is the most likely explanation of the observed pattern of numerous overlapping B-type reads [14,32]. Indeed, systematic analysis of the raw reads suggested that a long insertion (approximately 740 bases) in the 3′ flanking region near the end of the protein-coding region explains the observed B-type reads pattern (Supplementary file Figure S2).

**Figure 3:**
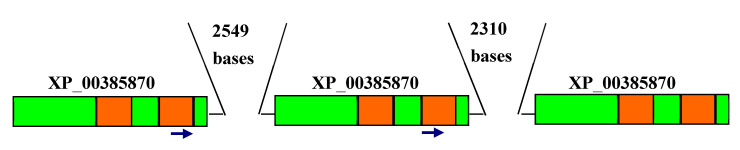
Schematic representation of regions of high and low similarity between XP_003858707.1, XP_003858708.1 and XP_003858709.1 sequences. Details are shown in the Supplementary Figure S3. Low similarity regions (less than 25% identity) are shown in green and high similarity regions (over 75% identity) are shown in red. Blue arrows represent two detected B-type reads.

**Figure 4:**
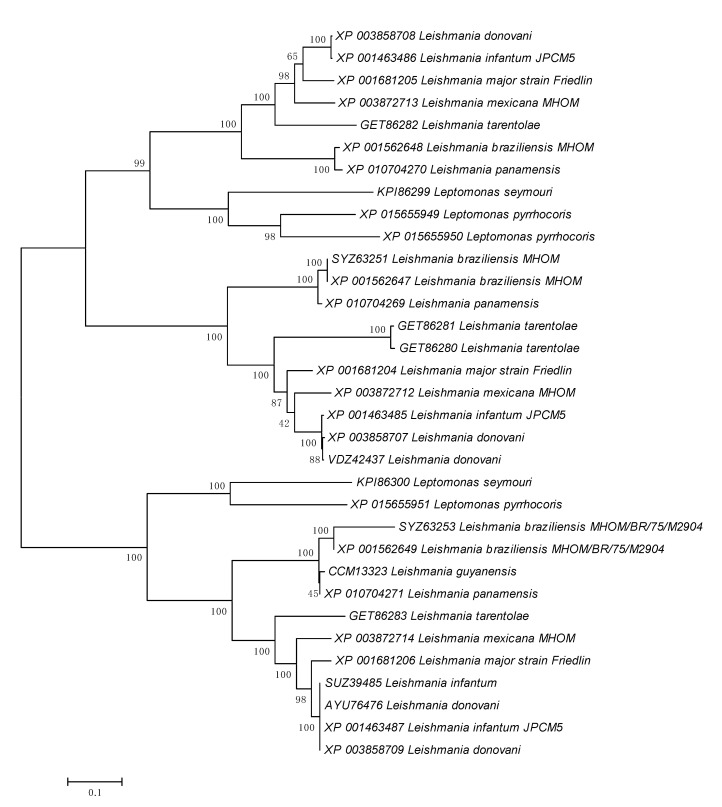
Maximum-likelihood phylogeny for the complete sequence alignment of the adenylate cyclase protein family. GenBank accession numbers precede species/isolate designations. The tree is drawn to scale, the scale bar denotes the number of substitutions per site. The bootstrap values (1000 replicates) are shown at the nodes.

Next we performed BLASTP searches of 137 protein-coding genes that overlap with such B-type reads (Supplementary file Table S2) against all proteins from the *Leishmania donovani* genome. Each BLASTP output was analyzed manually. Some of these protein-coding genes represent multigene families. An example of such a family is a putative adenylate cyclase protein (XP_003858707.1, XP_003858708.1, XP_003858709.1; the protein annotation was taken from a GenBank description of the homolog KPI87396; the BLASTP output is shown in the Supplementary Figure S3). One B-type read was found in the gene encoding the XP_003858707.1 sequence and another B-type read was found in gene encoding the XP_003858708.1 sequence (Figure 3 and Supplementary file Table S2). We extracted members of this protein family in other representatives of the subfamily Leishmaniinae [36]. A reconstructed phylogenetic tree suggested a complex evolutionary history of this multigene family, exemplified by the presence of multiple paralogs in the analyzed genomes (Figure 4).

**Figure 5:**
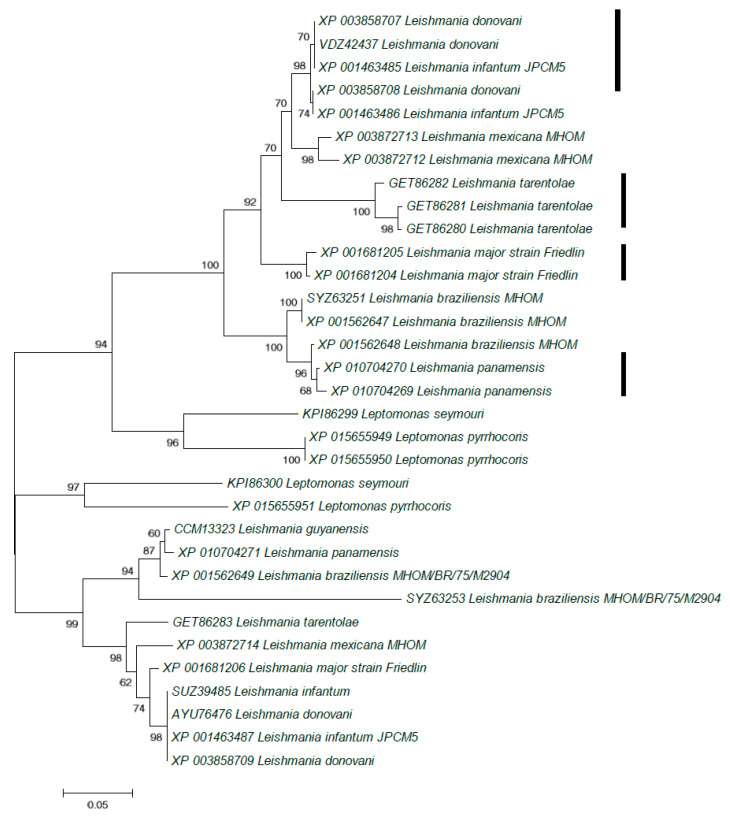
Maximum-likelihood phylogeny for two highly similar regions (positions 457–625 and 1114–1212) of the adenylate cyclase proteins. GenBank accession numbers precede species/isolate designations. The tree is drawn to scale, the scale bar denotes the number of substitutions per site. The bootstrap values (1000 replicates) are shown at the nodes. Major deviations (putative gene conversion events) from the tree inferred from the complete sequence alignment (Figure 4) are marked by black lines.

We also performed a phylogenetic analysis of two regions of unusually high conservation (XP_003858708.1 positions 336–510 and 721–806, Figure 3 and Supplementary file Figure S3). Analysis of the reconstructed phylogenetic tree (Figure 5) detected a group of nearly identical *Leishmania donovani* paralogous genes, this group was expected due to the choice of two analyzed regions (Figure 3 and Supplementary file Figure S3). In addition, three species-specific groups of nearly identical sequences from *L. tarentolae*, *L. major* and *L. panamensis* have been documented (Figure 5). Such tight species-specific clusters are likely to be the result of recurrent gene conversion events [37,38,39]. Of note, the vast majority of these adenylate cyclase genes form tandemly arranged paralogous gene clusters which may promote gene conversion events (as evident from the TriTryp.DB IDs: *L. donovani* BPK282A1: LdBPK_090280.1, LdBPK_090290.1, LdBPK_090300.1; *L. tarentolae*: LtaP09.0320, LtaP09.0330, LtaP19.0800, LtaP30.0010; *L. major* Friedlin: LmjF.09.0320, LmjF.09.0330, LmjF.09.0340; *L. panamensis* MHOM/COL/81/L13: LPAL13_090008100, LPAL13_090008200, LPAL13_090008300; all TriTryp.DB names are listed in the Supplementary file Table S3). The detected four species-specific groups of nearly identical sequences (Figure 5) do not exist in the phylogenetic tree for the complete sequence alignment (Figure 4), suggesting that putative recurrent gene events are highly specific for the two regions of this multigene family (Figure 3).

### 2.4. Analysis of Sacharomyces cerevisiae

We found only a few filtered reads in the yeast dataset (Table 4). This result suggests that the employed filtering procedure produces results that cannot be explained by whole-genome sequencing artifacts, because sequencing procedures and platforms were highly similar for both studied species.

## 3. Discussion

Comparison of the *Saccharomyces cerevisiae* and *Leishmania donovani* datasets suggests that the observed excess of B-type reads in *L. donovani* is not the result of sequencing/mapping artifacts. We propose that this excess is due to the recombination events. At least some of these events are likely to be gene conversion between members of multigene families, as illustrated by the example of the adenylate cyclase proteins (Figure 4 and Figure 5). This frequent recombination events are consistent with the recent study [32], which revealed greater genetic diversity, including extensive structural variation, than was previously suggested by geographically-focused studies [40]. It should be noted that there is an important methodological difference between our and previously published studies—the filtering procedures used in the latter were rather strict. Removal of these filters uncovered a substantial genomic variability of *Leishmania* isolates (Table 1). However, the usage of the strict filters is a justified and correct approach in order to estimate genetic distances between various samples.

Genetic recombination involves classical crossing-over and gene conversion. Polarity is one of the properties of gene conversion: in many cases the frequency of gene conversion exhibits a gradient across the gene monitored [33,34,35,41,42,43]. The frequency of conversion is usually dependent on its location. An interpretation of conversion polarity is that it is caused by the existence of specific initiation sites for recombination, located at the high end of the polarity gradient [34,43,44]. Here we show that the polarity gradient for the studied *L. donovani* is high at the 3′ end of the gene, implying that promoters of protein-coding genes less frequently contain initiation sites compared to the 3′ ends. An example of a putative gene conversion event is shown in Figure 3, where almost identical regions are located in the second half of the protein alignment. Gene conversion was observed in several Leishmaniinae species (Figure 5), implying a high frequency of these events. A substantially higher frequency of B-type reads compared to D-type reads (Table 1) is likely to be explained by the expected higher frequency of intrachromosomal gene conversion/recombination in the protein coding genes compared to interchromosomal gene conversion/recombination events. This is likely to be due to the presence of tandemly arranged multigene families that is a well-known property of the *L. donovani* genome [40].

The functional importance of so-called "concerted evolution" (frequent gene conversion events and unequal crossing-over) remains obscure [45]. For example, it appears that the rate of unequal crossing-over is much higher for rodent polyubiquitin genes than for their human kin, although there is no doubt that the function and conservation of these genes remain exactly the same during the evolution of mammals [46]. Proponents of the concerted evolution hypothesis suggest that the concerted pattern of fixation permits the establishment of biological novelty and species discontinuities in a manner not predicted by the classical genetics that is largely based on concepts of natural selection and genetic drift [47]. However the functional importance of frequent gene conversion events is still an important evolutionary question and systematic analysis of these events in *Leishmania* spp. may help to answer it. 

This is further exemplified by the case of trypanosomatid receptor adenylate cyclases [21], which are predicted to govern parasite–host interactions [48]. These proteins are extremely well studied in *Trypanosoma brucei*, where they have been implicated in *quorum sensing* regulation of differentiation in this species [49]. Some members of this protein family negatively regulate social motility in the procyclic stage of the trypanosome life cycle [50]. Nevertheless, the role of these proteins in *Leishmania* biology is under researched. Their expression was documented to be restricted to the sandfly-dwelling promastigotes in the case of *L. donovani* [51]. Our finding that gene conversion may have shaped the repertoire of these receptor proteins in different *Leishmania* spp. testifies to their importance and warrants future investigations into their functional role(s). 

## 4. Materials and Methods 

### 4.1. Datasets

We studied DNA-seq data for *Leishmania donovani* and *Saccharomyces cerevisiae*. For *L. donovani*, we used complete genomic data for the isolate BPK282A1 [52]. Reads were downloaded from the European Nucleotide Archive (ENA) (www.ebi.ac.uk/ena/data/view/PRJEB2086, 30 genomes). In the original study [40] the reference genome was masked at regions that were repetitive, duplicated, close to contig edges, structurally variable, or potentially mis-assembled. Five criteria masked a total of 6,358,203 bases out of the 32,444,998 bases reference genome sequence for *L. donovani* BPK282, resulting in SNPs (single-nucleotide polymorphisms) being called at 26,086,795 or 80.4% of the total nuclear genome [53]. We did not implement any of those filters because we were interested in the most dynamic fraction of the genome. For *S. cerevisiae*, we used DNA-seq data for isolate S288C (assembly R64, www.ncbi.nlm.nih.gov/genome/15). Read data are available under study ERP000140 at the ENA (www.ebi.ac.uk/ena/data/view/SRX155705, 10 genomes). In both cases, sequence reads were generated using the same Illumina HiSeq 2000 platform and standard protocols.

### 4.2. Data Binning and Filtering

We used the SMALT program (www.sanger.ac.uk/science/tools/smalt-0) for the mapping of paired reads. Firstly, we indexed the reference genomic sequences (ref_genome): smalt_x86_64 index -k 20 -s 13 ref_genome ref_genome.fas and then used paired reads (sampl1 and sample2) with the following set of parameters: smalt_x86_64 map -n 8 -f cigar -o output_file ref_genome sample1.fastq sample2.fastq 

We analyzed five SMALT mapping configurations (A/B/S/C/D-types): (A-type) mates are in proper orientation within the limits specified by the -i and -j options (the control set of properly aligned reads, i = 500 and j = 0); (B-type) mates in proper orientation outside the limits specified by the -i and -j options, but on the same chromosome (putative intra-chromosomal gene conversion events and sequencing errors); (C-type) mates are not in proper orientations, but on the same chromosome (mostly sequencing errors and inversion events that are known to be rare); (D-type) mates are mapped to different chromosomes (putative inter-chromosomal gene conversion events and sequencing errors); (S-type) a read was mapped as a single read (sole mapped read of a pair, sequencing errors although this type of errors may be different from other configurations). Manual inspection of the B-type reads suggested that two samples were obvious outliers (Supplementary Table S1). They were removed from further analysis.

We applied only one filter. We removed all reads with 95% identity, min 60 bases ungapped region; the percent identity for a B-type (or C-, or D-, or S-type) read should be equal or more 95% and more than the best overlapping A type read (the minimal mapped ungapped region ≥60 bases for all reads). This filter effectively removed errors of whole-genome sequencing procedures. All types of reads were analyzed as single reads (for example, if one B-type read was filtered out and its mate was not filtered, the second read was included in further analyses). After filtering, all overlapping reads were merged. If a merged set of reads (a merged read) overlapped with a known protein-coding sequence, we assigned this merged read to the set of reads that overlap with protein coding genes. We used the 2-tail Fisher exact test to evaluate homogeneity of 2 × 2 contingency tables [54]. A modified χ^2^ test was used for analyses of 2 × 3 contingency tables [54].

### 4.3. Phylogenetic Analysis

Evolutionary analyses were conducted in the package MEGA X [55] as described previously [56]. The phylogenies were inferred using the Maximum Likelihood method. The “Find Best Model (ML)” function was used to determine the appropriate substitution model. The model with the lowest Bayesian Information Criterion score was considered to best describe the substitution pattern for that dataset and was subsequently chosen for phylogenetic analysis. Initial trees for the heuristic search were obtained automatically by applying the Neighbor-Join and BioNJ algorithms to a matrix of pairwise distances estimated using a JTT model, and then selecting the topology with superior log likelihood value. A discrete Gamma distribution was used to model evolutionary rate differences among sites (2 categories (+G, parameter = 1.1990)). All positions with less than 90% site coverage were eliminated. That is, fewer than 10% alignment gaps, missing data, and ambiguous bases were allowed at any position. The multiple sequence alignment is presented in Supplementary file Figure S4.

## Figures and Tables

**Table 1:** The numbers and fractions of filtered reads in *Leishmania donovani* (28 samples). The total number of unfiltered A-type reads is 459,792,248.

Read Types	Overlap with Protein-Coding Region (Fraction)	No Overlaps with Protein-Coding Region (Fraction)
B	489 (35%)	927 (65%)
C	117 (27%)	308 (73%)
D	125 (21%)	473 (79%)
S	513 (36%)	901 (64%)

**Table 2:** Pairwise comparisons of different types of filtered reads in *Leishmania donovani.* The 2-tail Fisher exact test was used.

Read Types	C	D	S
**B**	0.0068	6.9 × 10^–10^	0.3460
**C**		0.0168	0.0009
**D**			5.3 × 10^−12^

**Table 3:** The number of filtered reads with numerous mismatches (the similarity level 90–95%).

Read Types	Overlap with Protein-Coding Region	No Overlaps with Protein-Coding Region
B	1	21
C	1	12
D	2	17
S	14	45

**Table 4:** The number of filtered reads in *Saccharomyces cerevisiae* (10 samples). The total number of unfiltered A-type reads is 158,344,992.

Read Types	Overlap with Protein-Coding Region	No Overlaps with Protein-Coding Region
B	0	0
C	0	3
D	1	0
S	0	1

## Supplementary Materials

The following are available online at https://www.mdpi.com/2076-0817/9/7/572/s1, Table S1: *L. donovani* DNA-seq samples, Table S2: 137 *L. donovani* protein-coding genes that overlap with merged B-type reads, Table S3: TriTryp.DB names of the putative adenylate cyclase proteins (Figure 4 and Figure 5), Figure S1: A BLASTP output for the XP_003861613.1 protein sequence (the multiple alignment format), Figure S2: Schematic representation of an insertion in the 3′ flanking region near the end of the gene encoding XP_003861613.1. Figure S3: A BLASTP output showing two conserved regions in XP_003858707.1, XP_003858708.1 and XP_003858709.1 protein sequences, Figure S4: Multiple sequence alignment used in this study.
