# Supplementary material for: Frequent Recombination Events in Leishmania donovani: Mining Population Data
Source: Pathogens. 2020 Jul 15;9(7):572. doi: 10.3390/pathogens9070572 (PMC7400496; doi:10.3390/pathogens9070572)
Supplement: Supplementary file 1 [file pathogens-09-00572-s001.pdf]

Frequent Recombination Events in Leishmania donovani: Mining Population Data

Igor B. Rogozin, Arzuv Charyyeva, Ivan A. Sidorenko, Vladimir N. Babenko and Vyacheslav Yurchenko

Supplementary Tables and Figures

Table S1: L. donovani DNA-seq samples (statistics of B-type reads).
CDS+ = B-type reads overlap with protein-coding genes
CDS- = B-type reads do not overlap with protein-coding genes

STUDIED SAMPLES (28):

Sample name	CDS+	CDS-
18830	8	148
18831	0	38
18833	0	14
18850	138	1597
18851	56	546
18852	50	552
18856	154	1207
18858	137	1280
18860	53	723
18861	29	743
18862	0	103
206260	115	1071
206261	2	1067
206263	9	471
206270	0	687
206271	67	936
206273	45	564
206275	3	708
206282	14	625
206284	92	947
206285	0	15
206286	11	679
206287	0	35
206288	0	42
206289	0	43
206290	8	109
206291	2	59
206292	0	15
Merged reads	166	1236

OUTLIERS (2):

Sample name	CDS+	CDS-
206271	4531	9662
206283	4336	9968
Merged reads	2572	3588

Supplementary Table S2. 137 L. donovani protein-coding genes that overlap with merged B-type reads.
ChromosomeID = the last three digits of the LOCUS number (NC_018***)
Number of merged reads = number of merged overlapping B-type reads
Match = number of matches in the longest merged read
L = the length of the the longest merged read
F = percent identity (Match/L)

Positions on Chromosome		Chromo-someID	#merged reads	Match	L	F	CDS positions	Locus_tag	protein product	status	protein_id
61398	61538	253	16	75	75	1	C(61468..64851)	LDBPK_260240	hypothetical protein	conserved	XP_003861613.1
690030	690154	258	13	72	75	0.96	C(689721..692840)	LDBPK_311500	hypothetical protein	unknown	XP_003863241.1
202748	202906	242	12	72	75	0.96	198985..204321	LDBPK_150550	hypothetical protein	conserved	XP_003859580.1
336257	336417	252	12	72	75	0.96	335255..336277	LDBPK_250930	hypothetical protein	unknown	XP_003861423.1
200249	200408	242	11	72	75	0.96	198985..204321	LDBPK_150550	hypothetical protein	conserved	XP_003859580.1
644053	644210	259	9	72	75	0.96	C(642591..644894)	LDBPK_321700	hypothetical protein	conserved	XP_003863586.1
727625	727736	247	9	72	75	0.96	C(726932..727834)	LDBPK_201710	hypothetical protein	conserved	XP_003860511.1
189492	189636	242	8	72	75	0.96	188745..191753	LDBPK_150520	katanin-like protein		XP_003859577.1
116492	116615	241	7	75	75	1	115139..116629	LDBPK_140370	hypothetical protein	conserved	XP_003859409.1
118037	118148	249	7	72	75	0.96	C(117509..120778)	LDBPK_220210	hypothetical protein	unknown	XP_003860771.1
1243529	1243609	258	7	72	75	0.96	C(1242582..1244072)	LDBPK_312550	hypothetical protein	conserved	XP_003863342.1
625518	625683	255	6	72	75	0.96	C(625597..627465)	LDBPK_281760	hypothetical protein	conserved	XP_003862275.1
496911	497046	242	6	75	75	1	C(496744..497028)	LDBPK_151240	nucleoside transporter 1		XP_003859646.1
639581	639676	258	6	72	75	0.96	C(639114..646298)	LDBPK_311440	hypothetical protein	conserved	XP_003863235.1
438125	438260	260	6	75	75	1	435816..439961	LDBPK_331230	hypothetical protein	conserved	XP_003863949.1
498646	498747	257	6	72	75	0.96	498149..498673	LDBPK_301480	ama1 protein	putative	XP_003862861.1
273558	273662	230	5	72	75	0.96	C(269424..278087)	LDBPK_030680	hypothetical protein	conserved	XP_003858033.1
491237	491455	253	5	72	75	0.96	490443..493889	LDBPK_261410	hypothetical protein	conserved	XP_003861728.1
521516	521607	237	5	72	75	0.96	520477..521577	LDBPK_101360	hypothetical protein	conserved	XP_003858969.1
301833	301966	231	5	71	75	1	C(301826..303757)	LDBPK_040740	hypothetical protein	conserved	XP_003858136.1
1101774	1101864	258	4	72	75	0.96	C(1100850..1102613)	LDBPK_312260	hypothetical protein		XP_003863314.1
1101930	1102005	258	4	72	75	0.96	C(1100850..1102613)	LDBPK_312260	hypothetical protein		XP_003863314.1
1285971	1286062	258	4	72	75	0.96	C(1285526..1286704)	LDBPK_312690	hypothetical protein	conserved	XP_003863356.1
144751	144850	262	4	72	75	0.96	140177..145222	LDBPK_350480	hypothetical protein	conserved	XP_003864641.1
230883	230980	252	4	75	75	1	C(228767..233809)	LDBPK_250690	myosin heavy chain kinase c-like		XP_003861399.1
152349	152485	239	4	72	75	0.96	150681..152351	LDBPK_120300	hypothetical protein	conserved	XP_003859147.1
666719	666873	255	4	73	75	1	C(666186..673556)	LDBPK_281830	hypothetical protein	conserved	XP_003862282.1
331661	331772	244	4	74	75	1	C(331662..333083)	LDBPK_170770	protein kinase	putative	XP_003859931.1
1102345	1102422	258	3	72	75	0.96	C(1100850..1102613)	LDBPK_312260	hypothetical protein		XP_003863314.1
327745	327869	239	3	72	75	0.96	322694..328441	LDBPK_120540	hypothetical protein	conserved	XP_003859171.1
1231460	1231547	257	3	75	75	1	1231356..1232528	LDBPK_303380	hypothetical protein	conserved	XP_003863049.1
1090454	1090613	256	3	72	75	0.96	1088835..1091009	LDBPK_292550	hypothetical protein	conserved	XP_003862673.1
496777	496867	242	3	75	75	1	C(496744..497028)	LDBPK_151240	nucleoside transporter	putative	XP_003859646.1
265863	265967	232	3	75	75	1	262729..266835	LDBPK_050720	phophatase-like protein		XP_003858259.1
908961	909042	259	3	72	75	0.96	C(908678..910600)	LDBPK_322440	hypothetical protein	conserved	XP_003863657.1
194100	194197	244	3	72	75	0.96	C(193248..198839)	LDBPK_170490	hypothetical protein	conserved	XP_003859903.1
269649	269799	235	3	72	75	1	263876..270697	LDBPK_080640	hypothetical protein	unknown	XP_003858629.1
436419	436512	237	3	72	75	1	C(436480..436947)	LDBPK_101050	histone H3		XP_003858939.1
124134	124217	243	3	72	75	0.96	C(123927..125372)	LDBPK_160380	hypothetical protein	conserved	XP_003859726.1
225374	225467	239	3	75	75	1	C(224123..225952)	LDBPK_120440	hypothetical protein	unknown	XP_003859161.1
85510	85605	238	3	72	75	0.96	83318..88723	LDBPK_110310	hypothetical protein	conserved	XP_003859008.1
689202	689287	263	2	72	75	0.96	C(679469..692572)	LDBPK_361800	hypothetical protein	conserved	XP_003865295.1
1560393	1560471	261	2	72	75	1	1560458..1562953	LDBPK_343800	adaptor gamma-1 chain	putative	XP_003864539.1
323359	323433	239	2	72	75	0.96	322694..328441	LDBPK_120540	hypothetical protein	conserved	XP_003859171.1
703969	704061	256	2	72	75	0.96	C(702923..704941)	LDBPK_291600	hypothetical protein	conserved	XP_003862579.1
1455467	1455541	261	2	75	75	1	1455165..1455716	LDBPK_343500	hypothetical protein	conserved	XP_003864509.1
742017	742098	255	2	72	75	0.96	C(740223..744005)	LDBPK_282000	hypothetical protein	conserved	XP_003862299.1
742492	742578	254	2	72	75	1	C(742346..744922)	LDBPK_271790	endo/exonuclease Mre11	putative	XP_003862030.1
2007744	2007824	262	2	72	75	0.96	2006915..2007775	LDBPK_355110	hypothetical protein	unknown	XP_003865088.1
2013376	2013451	262	2	72	75	0.96	2012852..2013772	LDBPK_355130	hypothetical protein	conserved	XP_003865090.1

243894	243997	261	2	72	75	0.96	243991..247728	LDBPK_340610	hypothetical protein	conserved	XP_003864226.1
191796	191872	258	2	72	75	0.96	C(190499..192532)	LDBPK_310550	hypothetical protein	unknown	XP_003863145.1
143706	143789	232	2	72	75	0.96	141177..143738	LDBPK_050440	hypothetical protein	conserved	XP_003858231.1
242630	242712	230	2	72	75	0.96	242690..245086	LDBPK_030640	hypothetical protein	conserved	XP_003858029.1
910217	910294	259	2	72	75	0.96	C(908678..910600)	LDBPK_322440	hypothetical protein	conserved	XP_003863657.1
460821	460899	244	2	72	75	0.96	458563..479592	LDBPK_171090	hypothetical protein	conserved	XP_003859963.1
625713	625789	255	2	72	75	0.96	C(625597..627465)	LDBPK_281760	hypothetical protein	conserved	XP_003862275.1
2008999	2009075	262	2	72	75	0.96	2008138..2010021	LDBPK_355120	biopterin transporter	putative	XP_003865089.1
181610	181684	228	2	75	75	1	180454..182241	LDBPK_010640	hypothetical protein	conserved	XP_003857877.1
981481	981569	253	2	72	75	0.96	980998..982443	LDBPK_262540	protein kinase	putative	XP_003861838.1
239126	239234	240	2	72	75	0.96	C(238081..240228)	LDBPK_130670	protein kinase	putative	XP_003859278.1
974465	974540	258	2	72	75	0.96	C(974451..974765)	LDBPK_312010	tryparedoxin-like protein		XP_003863290.1
1163191	1163269	258	2	72	75	0.96	C(1162333..1163382)	LDBPK_312400	3,2-trans-enoyl-CoA isomerase, mt		XP_003863328.1
878073	878154	257	2	72	75	0.96	C(877746..881210)	LDBPK_302350	zinc-finger protein	conserved	XP_003862947.1
189708	189784	242	2	73	75	1	188745..191753	LDBPK_150520	katanin-like protein		XP_003859577.1
22522	22597	254	2	72	75	0.96	C(20913..22691)	LDBPK_270090	cytochrome p450-like protein		XP_003861867.1
204904	204972	252	2	69	69	1	C(204904..206916)	LDBPK_250620	hypothetical protein	conserved	XP_003861392.1
610376	610446	245	1	68	71	0.958	608633..611323	LDBPK_181400	pumilio protein	putative	XP_003860154.1
509853	509927	256	1	72	75	0.96	504828..510272	LDBPK_291340	hypothetical protein	unknown	XP_003862551.1
160501	160575	261	1	72	75	0.96	159562..161775	LDBPK_340480	hypothetical protein	unknown	XP_003864213.1
1681477	1681551	261	1	72	75	0.96	1680767..1681939	LDBPK_344190	RNA-binding protein-like protein		XP_003864579.1
486553	486627	236	1	72	75	0.96	C(486555..487817)	LDBPK_091270	hypothetical protein	unknown	XP_003858805.1
285076	285150	230	1	72	75	0.96	C(284993..286207)	LDBPK_030710	hypothetical protein	conserved	XP_003858036.1
1493124	1493198	259	1	72	75	0.96	1492688..1493563	LDBPK_324000	hypothetical protein	unknown	XP_003863811.1
887542	887616	256	1	72	75	0.96	C(885595..891426)	LDBPK_292100	hypothetical protein	conserved	XP_003862628.1
158357	158431	231	1	72	75	0.96	C(147591..159950)	LDBPK_040410	hypothetical protein	conserved	XP_003858104.1
1664079	1664153	261	1	75	75	1	1664014..1664160	LDBPK_344150	hypothetical protein	conserved	XP_003864574.1
1456356	1456418	263	1	63	63	1	1456117..1456377	LDBPK_363940	40S ribosomalS 27-1	putative	XP_003865508.1
692288	692362	263	1	75	75	1	C(679469..692572)	LDBPK_361800	hypothetical protein	conserved	XP_003865295.1
186830	186895	252	1	66	66	1	C(186420..187313)	LDBPK_250550	hypothetical protein	conserved	XP_003861385.1
940102	940176	256	1	72	75	0.96	C(936801..941609)	LDBPK_292210	hypothetical protein	conserved	XP_003862639.1
401489	401563	234	1	72	75	0.96	399386..401572	LDBPK_070970	hypothetical protein	conserved	XP_003858533.1
339534	339604	230	1	71	71	1	C(337892..340801)	LDBPK_013360	uncharacterized protein		XP_003858049.1
682825	682899	255	1	72	75	0.96	C(682700..684466)	LDBPK_281860	hypothetical protein	conserved	XP_003862285.1
487149	487223	234	1	72	75	0.96	481591..489309	LDBPK_071070	hypothetical protein	conserved	XP_003858543.1
440749	440823	244	1	72	75	0.96	438971..443749	LDBPK_171060	hypothetical protein	conserved	XP_003859960.1
211403	211477	242	1	72	75	0.96	209289..214544	LDBPK_150570	hypothetical protein	unknown	XP_003859582.1
268424	268498	246	1	72	75	0.96	268110..270443	LDBPK_190600	hypothetical protein	unknown	XP_003860238.1
1291806	1291880	263	1	72	75	0.96	C(1291880..1294723)	LDBPK_363350	DNA topoisomerase III	putative	XP_003865450.1
533670	533732	259	1	63	63	1	533220..536534	LDBPK_321420	RNA guanylyltransferase	putative	XP_003863558.1
308957	309017	229	1	61	61	1	308978..309556	LDBPK_020650	hypothetical protein	conserved	XP_003857960.1
147359	147433	241	1	72	75	0.96	146129..148582	LDBPK_140430	hypothetical protein	conserved	XP_003859415.1
280616	280690	252	1	72	75	0.96	280399..281319	LDBPK_250810	hypothetical protein	unknown	XP_003861411.1
934506	934580	253	1	72	75	0.96	926370..941693	LDBPK_262400	hypothetical protein	conserved	XP_003861825.1
124863	124937	237	1	72	75	0.96	122945..125242	LDBPK_100320	hypothetical protein	conserved	XP_003858871.1
886959	887033	261	1	72	75	0.96	C(885143..890974)	LDBPK_342010	hypothetical protein	conserved	XP_003864361.1
460543	460617	244	1	72	75	0.96	458563..479592	LDBPK_171090	hypothetical protein	conserved	XP_003859963.1
473883	473957	239	1	72	75	0.96	470751..474983	LDBPK_120065	uncharacterized protein		XP_003859191.1
151544	151618	229	1	72	75	0.96	C(147597..152216)	LDBPK_020300	glycosyl transferase,	putative	XP_003857926.1
227167	227241	235	1	72	75	0.96	226751..227788	LDBPK_080570	translation initiation factor-like		XP_003858622.1
302757	302831	263	1	72	75	0.96	299919..305729	LDBPK_070760	hypothetical protein	conserved	XP_024329263.1
536222	536296	242	1	72	75	0.96	C(534562..537066)	LDBPK_151330	MGT1 magnesium transporter		XP_003859656.1
467129	467203	257	1	72	75	0.96	466158..468299	LDBPK_301410	GTPase activating	putative	XP_003862854.1
939991	940065	256	1	72	75	0.96	C(936801..941609)	LDBPK_292210	hypothetical protein	conserved	XP_003862639.1
151255	151327	239	1	73	73	1	150681..152351	LDBPK_120300	hypothetical protein	conserved	XP_003859147.1
778765	778839	254	1	72	75	0.96	C(777632..778924)	LDBPK_271880	hypothetical protein	conserved	XP_003862039.1
251689	251763	262	1	72	75	0.96	251096..254491	LDBPK_350560	phosphatidylinositol-4-phosphate 5-kinase-like		XP_003864644.1
703840	703914	256	1	72	75	0.96	C(702923..704941)	LDBPK_291600	hypothetical protein	conserved	XP_003862579.1

471185	471259	244	1	72	75	0.96	458563..479592	LDBPK_171090	hypothetical protein	conserved	XP_003859963.1
395888	395962	259	1	72	75	0.96	395690..396919	LDBPK_321040	hypothetical protein	conserved	XP_003863520.1
366716	366790	241	1	75	75	1	C(366044..369307)	LDBPK_140940	hypothetical protein	conserved	XP_003859461.1
289708	289782	246	1	72	75	0.96	287482..293280	LDBPK_190650	hypothetical protein	conserved	XP_003860243.1
122258	122318	244	1	61	61	1	C(121144..123501)	LDBPK_170310	hypothetical protein	conserved	XP_003859885.1
279785	279859	237	1	72	75	0.96	C(277712..280978)	LDBPK_100590	hypothetical protein	unknown	XP_003858895.1
231039	231113	252	1	72	75	0.96	C(228767..233809)	LDBPK_250690	myosin heavy chain kinase c-like		XP_003861399.1
313180	313251	232	1	72	72	1	311613..313616	LDBPK_050850	hypothetical protein	conserved	XP_003858272.1
170521	170595	232	1	72	75	0.96	169396..175689	LDBPK_050530	kinetoplast-associated protein like		XP_003858240.1
267648	267712	242	1	65	65	1	265998..268094	LDBPK_150680	hypothetical protein	unknown	XP_003859593.1
534778	534852	257	1	72	75	0.96	533581..536754	LDBPK_301540	hypothetical protein	conserved	XP_003862867.1
339626	339700	232	1	72	75	0.96	339118..342384	LDBPK_050920	paraflagellar rod	putative	XP_003858279.1
738240	738314	258	1	72	75	0.96	C(735944..738772)	LDBPK_311550	hypothetical protein	conserved	XP_003863246.1
467887	467961	241	1	72	75	0.96	467861..469057	LDBPK_141150	hypothetical protein	conserved	XP_003859482.1
1163338	1163412	258	1	72	75	0.96	C(1162333..1163382)	LDBPK_312400	3,2-trans-enoyl-CoA isomerase		XP_003863328.1
216852	216926	236	1	72	75	0.96	213108..217625	LDBPK_090550	hypothetical protein	conserved	XP_003858735.1
123089	123163	228	1	72	75	0.96	119408..123727	LDBPK_010480	hypothetical	conserved	XP_003857861.1
664004	664078	245	1	72	75	0.96	663947..664093	LDBPK_181490	P-type H+-ATPase,	putative	XP_003860163.1
152401	152475	261	1	72	75	0.96	152165..152527	LDBPK_340460	ribosomal protein S25		XP_003864211.1
372921	372995	258	1	72	75	0.96	C(372754..374013)	LDBPK_310990	hypothetical protein	unknown	XP_003863191.1
103073	103147	236	1	72	75	0.96	101296..103848	LDBPK_090290	hypothetical protein	conserved	XP_003858708.1
1285944	1286010	262	1	64	67	0.955	C(1283936..1286689)	LDBPK_353150	ATP-dependent RNA helicase putative		XP_003864894.1
920399	920474	255	1	76	76	1	919135..921849	LDBPK_282490	hypothetical protein	conserved	XP_003862347.1
984756	984830	254	1	75	75	1	983617..984900	LDBPK_020730	hypothetical protein	conserved	XP_003862098.1
108379	108444	251	1	66	66	1	107900..108451	LDBPK_240350	CMP-sialic acid transporter putative		XP_003861125.1
94925	94998	239	1	74	74	1	91189..99972	LDBPK_120260	hypothetical protein	conserved	XP_003859138.1
97601	97663	236	1	63	63	1	95989..98748	LDBPK_090280	hypothetical protein	conserved	XP_003858707.1
1092351	1092426	259	1	76	76	1	C(1090269..1092914)	LDBPK_322900	hypothetical protein	conserved	XP_003863703.1
71243	71306	236	1	61	64	0.953	71060..71464	LDBPK_090180	hypothetical protein	putative	XP_003858697.1

Supplementary Table S3: TriTryp.DB names of the putative adenylate cyclase proteins (Figures 4 and 5).

Refseq ID	TriTrypDB ID
XP_003858708	LdBPK_090290.1
XP_001463486	LINF_090008900
XP_001681205	LmjF.09.0330
XP_003872713	LmxM.09.0330
GET86282	LtaP19.0800
XP_001562648	LbrM.09.0320
XP_010704270	LPMP_090320
XP_015655949	LpyrH10_16_0770
XP_015655950	LpyrH10_16_0780
KPI86299	Lsey_0138_0140
XP_001463485	LINF_090008800
XP_003858707	LdBPK_090280.1
VDZ42437	LdBPK.09.2.000280.1
XP_003872712	LmxM.09.0320
XP_001681204	LmjF.09.0320
GET86281	LtaP30.0010
XP_001562647	LbrM.09.0310
XP_010704269	LPMP_090310
XP_001681206	LmjF.09.0340
XP_003872714	LmxM.09.0340
AYU76476	LdCL_090008700
XP_001463487	LINF_090009000
XP_003858709	LdBPK_090300.1
KPI86300	Lsey_0138_0150
XP_001562649	LbrM.09.0330
GET86283	LtaP09.0330
XP_010704271	LPMP_090330
XP_015655951	LpyrH10_16_0790

Supplementary Figure S1. A BLASTP output for the XP_003861613.1 protein sequence. The blue arrow at the end of the protein-coding region shows the merged B-type read (Supplementary Table S2).

```
XP_003861613.1  1    MDTSSSARYADQLSVVHLSHIHEGVLQLREHLRSALLDDSPQTEMIVYTRLYQLYRDFYLPFFVAKLHIPSQLPPGAGSA  80
AYU79624.1      1    MDTSSSARYADQLSVVHLSHIHEGVLQLREHLRSALLDDSPQTEMIVYTRLYQLYRDFYLPFFVAKLHIPSQLPPGAGSA  80
VDZ45478.1      1    MDTSSSARYADQLSVVHLSHIHEGVLQLREHLRSALLDDSPQTEMIVYTRLYQLYRDFYLPFFVAKLHIPSQLPPGAGSA  80
TPP41076.1      1    MDTSSSARYADQLSVVHLSHIHEGVLQLREHLRSALLDDSPQTEMIVYTRLYQLYRDFYLPFFVAKLHIPSQLPPGAGSA  80
TPP51924.1      1    MDTSSSARYADQLSVVHLSHIHEGVLQLREHLRSALLDDSPQTEMIVYTRLYQLYRDFYLPFFVAKLHIPSQLPPGAGSA  80

XP_003861613.1  81   PSPDARPAASAEARFVMTTYTQDVLAACVYLLDPASLPATYIVTSTPPACEEAQNEANASSVYISAEQRRFCGRVFLYMC  160
AYU79624.1      81   PSPDARPAASAEARFVMTTYTQDVLAACVYLLDPASLPATYIVTSTPPACEEAQNEANASSVYISAEQRRFCGRVFLYMC  160
VDZ45478.1      81   PSLDARPAASAEARFVMTTYTQDVLAACVYLLDPASLPATYIVTSTPPACEEAQNEANASSVYISAEQRRFCGRVFLYMC  160
TPP41076.1      81   PSPDARPAASAEARFVMTTYTQDVLAACVYLLDPASLPATYIVTSTPPACEEAQNEANASSVYISAEQRRFCGRVFLYMC  160
TPP51924.1      81   PSLDARPAASAEARFVMTTYTQDVLAACVYLLDPASLPATYIVTSTPPACEEAQNEANASSVYISAEQRRFCGRVFLYMC  160

XP_003861613.1  161  WASAALPLHIVLQALYPLLDPGVSGAPRATTTTLSTTADPPRERQGPSLTENHIAADLFSQERFACQKRLLRDTLNGLVL  240
AYU79624.1      161  WASAALPLHIVLQALYPLLDPGVSGAPRATTTTLSTTADPPRERQGPSLTENHIAADLFSQERFACQKRLLRDTLNGLVL  240
VDZ45478.1      161  WASAALPLHIVLQALYPLLDPGVSGAPRATTTTLSTTADPPRERQGPSLTENHIAADLFSQERFACQKRLLRDTLNGLVL  240
TPP41076.1      161  WASAALPLHIVLQALYPLLDPGVSGAPRATTTTLSTTADPPRERQGPSLTENHIAADLFSQERFACQKRLLRDTLNGLVL  240
TPP51924.1      161  WASAALPLHIVLQALYPLLDPGVSGAPRATTTTLSTTADPPRERQGPSLTENHIAADLFSQERFACQKRLLRDTLNGLVL  240

XP_003861613.1  241  TRANGVRALLRVLLLNDRVTTEMTGEAAQLLVQLLTSPVFTLWQQQQPQQLGESLGYRSPFSAAGEEECAAEVVVTHVTT  320
AYU79624.1      241  TRANGVRALLRVLLLNDRVTTEMTGEAAQLLVQLLTSPVFTLWQQQQPQQLGESLGYRSPFSAAGEEECAAEVVVTHVTT  320
VDZ45478.1      241  TRANGVRALLRVLLLNDRVTTEMTGEAAQLLVQLLTSPVFTLWQQQQPQQLGESLGYRSPFSAAGEEECAAEVVVTHVTT  320
TPP41076.1      241  TRANGVRALLRVLLLNDRVTTEMTGEAAQLLVQLLTSPVFT------------------PFSAAGEEECAAEVVVTHVTT  302
TPP51924.1      241  TRANGVRALLRVLLLNDRVTTEMTGEAAQLLVQLLTSPVFT------------------PFSAAGEEECAAEVVVTHVTT  302

XP_003861613.1  321  ALSVEDQVRLLAPQLLALLEEHADATLAVSSASRTAARPRFLARLDAARLRLPAETLEQRLHLALTMLLNALVRLPPRDK  400
AYU79624.1      321  ALSVEDQVRLLAPQLLALLEEHADATLAVSSASRTAARPRFLARLDAARLRLPAETLEQRLHLALTMLLNALVRLPPRDK  400
VDZ45478.1      321  ALSVEDQVRLLAPQLLALLEEHADATLAVSSASRTAARPRFLARLDAARLRLPAETLEQRLHLALTMLLNALVRLPPRDK  400
TPP41076.1      303  ALSVEDQVRLLAPQLLALLEEHADATLAVSSASRTAARPRFLARLDAARLRLPAETLEQRLHLALTMLLNALVRLPPRDK  382
TPP51924.1      303  ALSVEDQVRLLAPQLLALLEEHADATLAVSSASRTAARPRFLARLDAARLRLPAETLEQRLHLALTMLLNALVRLPPRDK  382

XP_003861613.1  401  HDFARSYRQLFYTNKFVLSPGFGCLSLRSDTDVVEDDAIAALLRLGSLIKGVSSGAGSGVMAHVLPATAAGLLNICAVLA  480
AYU79624.1      401  HDFARSYRQLFYTNKFVLSPGFGCLSLRSDTDVVEDDAIAALLRLGSLIKGVSSGAGSGVMAHVLPATAAGLLNICAVLA  480
VDZ45478.1      401  HDFARSYRQLFYTNKFVLSPGFGCLSLRSDTDVVEDDAIAALLRLGSLIKGVSSGAGSGVMAHVLPATAAGLLNICAVLA  480
TPP41076.1      383  HDFARSYRQLFYTNKFVLSPGFGCLSLRSDTDVVEDDAIAALLRLGSLIKGVSSGAGSGVMAHVLPATAAGLLNICAVLA  462
TPP51924.1      383  HDFARSYRQLFYTNKFVLSPGFGCLSLRSDTDVVEDDAIAALLRLGSLIKGVSSGAGSGVMAHVLPATAAGLLNICAVLA  462

XP_003861613.1  481  RSCDGDGNAAQFVSPSLPATLRSLFSELLSSPSLYDLCARALVNACGEARAHCYLPGNAAQPRLRYTRDVCGRERLVTGL  560
AYU79624.1      481  RSCDGDGNAAQFVSPSLPATLRSLFSELLSSPSLYDLCSRALVNACGEARAHCYLPGNAAQPRLRYTRDVCGRERLVTGL  560
VDZ45478.1      481  RSCDGDGNAAQFVSPSLPATLRSLFSELLSSPSLYDLCARALVNACGEARAHCYLPGNAAQPRLRYTRDVCGRERLVTGL  560
TPP41076.1      463  RSCDGDGNAAQFVSPSLPATLRSLFSELLSSPSLYDLCARALVNACGEARAHCYLPGNAAQPRLRYTRDVCGRERLVTGL  542
TPP51924.1      463  RSCDGDGNAAQFVSPSLPATLRSLFSELLSSPSLYDLCARALVNACGEARAHCYLPGNAAQPRLRYTRDVCGRERLVTGL  542

XP_003861613.1  561  QWLLLDVAATTPDFVHTCVDVMVEACHLELFAAGVENLTVSPAPRLTETKLLVSAPTTENDTSPLIALLERLSLEATPEA  640
AYU79624.1      561  QWLLLDVAATTPDFVHTCVDVMVEACHLELFAAGVENLTVSPAPRLTETKLLVSAPTTENDTSPLIALLERLSLEATPEA  640
VDZ45478.1      561  QWLLLDVAATTPDFVHTCVDVMVEACHLELFAAGVENLTVSPAPRLTETKLLVSAPTTENDTSPLIALLERLSLEATPEA  640
TPP41076.1      543  QWLLLDVAATTPDFVHTCVDVMVEACHLELFAAGVENLTVSPAPRLTETKLLVSAPTTENDTSPLIALLERLSLEATPEA  622
TPP51924.1      543  QWLLLDVAATTPDFVHTCVDVMVEACHLELFAAGVENLTVSPAPRLTETKLLVSAPTTENDTSPLIALLERLSLEATPEA  622

XP_003861613.1  641  LFGSRGASLASTLELLGRMLALSGALYRWALGLVEHLLAASSVEVHLGGGDTRAERWQNLSRLQRCSRKILASLTQLNTP  720
AYU79624.1      641  LFGSRGASLASTLELLGRMLALSGALYRWALGLVEHLLAASSVEVHLGGGDTRAERWQNLSRLQRCSRKILASLTQLNTP  720
VDZ45478.1      641  LFGSRGASLASTLELLGRMLALSGALYRWALGLVEHLLAASSVEVHFGGGDTRAERWQNLSRLQRCSRKILASLTQLNTP  720
TPP41076.1      623  LFGSRGASLASTLELLGRMLALSGALYRWALGLVEHLLAASSVEVHLGGGDTRAERWQNLSRLQRCSRKILASLTQLNTP  702
TPP51924.1      623  LFGSRGASLASTLELLGRMLALSGALYRWALGLVEHLLAASSVEVHFGGGDTRAERWQNLSRLQRCSRKILASLTQLNTP  702

XP_003861613.1  721  TPRGGAWHSVVSTDGAQLSLTARVQQALGAFLERIEEHLASCEAVEQLSYDKQHRQGEAITAVRAEWEQLAEKLRAALDS  800
AYU79624.1      721  TPRGGAWHSVVSTDGAQLSLTARVQQALGAFLERIEEHLASCEAVEQLSYDKQHRQGEAITAVRAEWEQLAEKLRAALDS  800
VDZ45478.1      721  TPRGGAWHSVVSTDGAQLSLTARVQQALGAFLERIEEHLASCEAVEQLSYDKQHRQGEAITAVRAEWEQLAEKLRAALDS  800
TPP41076.1      703  TPRGGAWHSVVSTDGAQLSLTARVQQALGAFLERIEEHLASCEAVEQLSYDKQHRQGEAITAVRAEWEQLAEKLRAALDS  782
TPP51924.1      703  TPRGGAWHSVVSTDGAQLSLTARVQQALGAFLERIEEHLASCEAVEQLSYDKQHRQGEAITAVRAEWEQLAEKLRAALDS  782
```

```
XP_003861613.1  801   RAAIDVAVTLAKLAHGVDDVVFEVTDPQPLYDTTQRLLQLLVRVLCEADDVGAAVRAVHCVAWLGMYRFDSKDSTYMADV  880
AYU79624.1      801   RAAIDVAVTLAKLAHGVDDVVFEVTDPQPLYDTTQRLLQLLVRVLCEADDVGAAVRAVHCVAWLGMYRFDSKDSTYMADV  880
VDZ45478.1      801   RAAIDVAVTLAKLAHGVDDVVFEVTDPQPLYDTTQRLLQLLVRVLFEADDVGAAVRAVHCVAWLGMYRFDSKDSTYMADV  880
TPP41076.1      783   RAAIDVAVTLAKLAHGVDDVVFEVTDPQPLYDTTQRLLQLLVRVLCEADDVGAAVRAVHCVAWLGMYRFDSKDSTYMADV  862
TPP51924.1      783   RAAIDVAVTLAKLAHGVDDVVFEVTDPQPLYDTTQRLLQLLVRVLFEADDVGAAVRAVHCVAWLGMYRFDSKDSTYMADV  862

XP_003861613.1  881   MWSVLAEDHLPPWLSASAEYTVATQAAAASRICRLRVRVLDVLLSWTDYDEDDLTLRNLDDSLRRKHHTSLYDVLVALCR  960
AYU79624.1      881   MWSVLAEDHLPPWLSASAEYTVATQAAAASRICRLRVRVLDVLLSWTDYDEDDLTLRNLDDSLRRKHHTSLYDVLVALCR  960
VDZ45478.1      881   MWSVLAEDHLPPWLSASAEYTVATQAAAASRICRLRVRVLDVLLSWTDYDEDDLTLHNLDDSLRRTHHTSLYDVLVALCR  960
TPP41076.1      863   MWSVLAEDHLPPWLSASAEYTVATQAAAASRICRLRVRVLDVLLSWTDYDEDDLTLRNLDDSLRRKHHTSLYDVLVALCR  942
TPP51924.1      863   MWSVLAEDHLPPWLSASAEYTVATQAAAASRICRLRVRVLDVLLSWTDYDEDDLTLHNLDDSLRRTHHTSLYDVLVALCR  942

XP_003861613.1  961   STEDAFVQVAALHFIGTYALAMQPRVPVSAICDLCRDVFRLSRHEMAKAACAAALGKIVASLCHPSDATLLVLSELDINN  1040
AYU79624.1      961   STEDAFVQVAALHFIGTYALAMQPRVPVSAICDLCRDVFRLSRHEMAKAACAAALGKIVASLCHPSDATLLVLSELDINN  1040
VDZ45478.1      961   STEDAFVQVAALHFIGTYALAMQPRVPVSAIFDLCRDVFRLSRHEMAKAACAAALGKIVASLCHPSDATLLVLSELDINN  1040
TPP41076.1      943   STEDAFVQVAALHFIGTYALAMQPRVPVSAICDLCRDVFRLSRHEMAKAACAAALGKIVASLCHPSDATLLVLSELDINN  1022
TPP51924.1      943   STEDAFVQVAALHFIGTYALAMQPRVPVSAIFDLCRDVFRLSRHEMAKAACAAALGKIVASLCHPSDATLLVLSELDINN  1022

XP_003861613.1  1041  LQTLASAMSSYRGRPHVEGNTSVTAPAMSAAAETDLHDEVIRLHGREMLAILRRTSLGFGGARAGAAPAAADDLRVSSLR  1120
AYU79624.1      1041  LQTLASAMSSYRGRPHVEGNASVTAPAMSAAAETDLHDEVIRLHGREMLAILRRTSLGFGGARAGAAPAAADDLRVSSLR  1120
VDZ45478.1      1041  LQTLASAMSSYRGRPHVEGNASVTAPAMSAAAETDLHDEVIRLHGREMLAILRRTSLGFGGARAGAAPAAADDLRVSSLR  1120
TPP41076.1      1023  LQTLASAMSSYRGRPHVEGNTSVTAPAMSAAAETDLHDEVIRLHGREMLAILRRTSLGFGGARAGAAPAAADDLRVSSLR  1102
TPP51924.1      1023  LQTLASAMSSYRGRPHVEGNASVTAPAMSAAAETDLHDEVIRLHGREMLAILRRTSLGFGGARAGAAPAAADDLRVSSLR  1102
                                                                                             --------

XP_003861613.1  1121  VSELRRA  1127
AYU79624.1      1121  VSELRRA  1127
VDZ45478.1      1121  VSELRRA  1127
TPP41076.1      1103  VSELRRA  1109
TPP51924.1      1103  VSELRRA  1109  3' flanking region
                      --------------------->
```

Supplementary Figure S2. Schematic representation of an insertion in the 3' flanking region near the end of the gene encoding the XP_003861613.1 protein. The gene is located in the complementary strand.

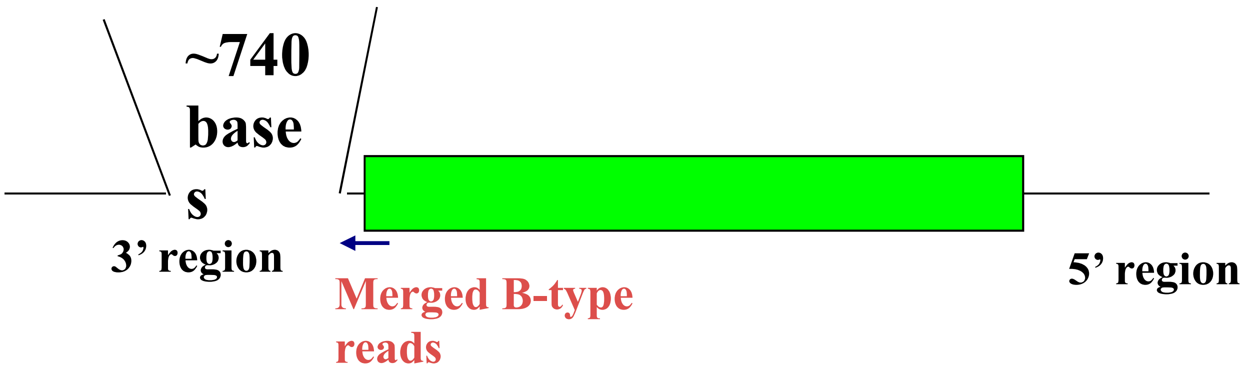

Supplementary Figure S3. A BLASTP output showing 2 conserved regions in the XP_003858708.1 protein sequence.

```
BLASTP 2.2.6 [Apr-09-2003]

Query= "XP_003858708.1"
         (850 letters)

Database: ldon.fa
           8014 sequences; 4,920,192 total letters

                                                                 Score    E
Sequences producing significant alignments:                      (bits) Value

XP_003858707.1 hypothetical protein, conserved [Leishmania donov...   675   0.0
XP_003858709.1 hypothetical protein, conserved [Leishmania donov...   342   3e-094


>XP_003858707.1 hypothetical protein, conserved [Leishmania donovani]
          Length = 919

 Score =  675 bits (1741), Expect = 0.0
 Identities = 412/814 (50%), Positives = 496/814 (60%), Gaps = 59/814 (7%)

Query: 26  YIGIVAFVACLLLIFVGIFSPLIVLRNMDKSYEAEMRYIEYTLANKAVAPYRVVMVEAFA 85
           YI     + C L IF+GI  P++V+R     + A +R  EY   +   +  R VM+E FA
Sbjct: 99  YIITACVLGCALTIFIGILVPMLVIRRSANIHNAVVRAKEYDAVSAWASVLRDVMIEGFA 158

Query: 86  ISQALEGYVMSRMLVMPNLSAP-------PLERIQGQNFTNFDDIGATLRRNTTMIAMTA 138
            ++AL GY +S     P L  P       P ++I       F    + +      +A+ A
Sbjct: 159 ATRALAGYSIS---TFPPLQTPTNASDSIPSDKITAY-LKRFPRFASLIASKKPAVAIQA 214

Query: 139 LQPGGVYAFFSPRNATKYGRDTLDPNDNAQNFKPTPFETILSGRAVYVGPFRSTSDVLRG 198
           + PGGV A   P +    GRD + P+D    +KP+  ET  SGR   VGP R+T   L+
Sbjct: 215 ICPGGVIAMTHPHDPDTVGRDLMSPSDPTNRYKPSTRETAQSGRYAIVGPRRTTIASLKQ 274

Query: 199 HWGLAIRRPIYNRTSYESADIR-TFWGFAFTLVNISGLIDKNPFAFTSLSKKRDVDYLLS 257
            W +  R P+Y  TS      + TFWGF   +VN++G +D        L+K  ++DY+L
Sbjct: 275 IWVIFTRAPLYRNTSKGVIPSQETFWGFVLLVVNVTGALDV--MDLDKLAKDHNLDYVLY 332

Query: 258 VHNETSGVITVLATSLKQPTATQLADFVRLGTSVGIT----PNHPFLLTIRGRDYGSNLS 313
             +  SG   V+A+SL  P+     D+        +T    P+    + +R R+   +L+
Sbjct: 333 DTSTESGDTHVIASSL--PSGAMQPDYEEFVAESTVTDVLAPHSSLYIAVRSRETYVSLT 390

Query: 314 PANIIIVVSTVLSGLLAIFAAVIAAVLWCTATYDAAAHAPKMAPFAMLTIGPCRGEELWD 373  Conserved
           P NII++V   L G L +    IAAVLWCTATYDAAAHAPKMAPFAMLTIGPCRGEELWD
Sbjct: 391 PTNIILIVVWTLFGSLLLLGISIAAVLWCTATYDAAAHAPKMAPFAMLTIGPCRGEELWD 450

Query: 374 LATDQMAEVTEKLDQVLVRQMERHRAYQIQQVHPLTTSYVTRSVAAAVQMAFSTIEELQR 433
           LATDQMAEVTEKLDQVLVRQMERHRAYQIQQVHPLTTSYVTRSVAAAVQMAFSTIEELQR
Sbjct: 451 LATDQMAEVTEKLDQVLVRQMERHRAYQIQQVHPLTTSYVTRSVAAAVQMAFSTIEELQR 510

Query: 434 HPIDGPLRAVLGDEVRLLLCYAVHWCTDAAVRVESLEGTYRYEGCDVVFGGRMWAFAAPS 493
           HPIDGPLRAVLGDEVRLLLCYAVHWCTDAAVRVESLEGTYRYEGCDVVFGGRMWAFAAPS
Sbjct: 511 HPIDGPLRAVLGDEVRLLLCYAVHWCTDAAVRVESLEGTYRYEGCDVVFGGRMWAFAAPS 570

Query: 494 VVTASEAVVQTLPCFGLRSIDTRPRCVVNVVRSRTLCGATGGKEL----------GGKSA 543  region #1
           VVTASEAVVQTLPCFGL  + TR    V+V+R+ T    T G E           GG SA
Sbjct: 571 VVTASEAVVQTLPCFGLSGVVTRAYQTVDVMRT-TRASYTDGSEAGSSDASSTIGGGGSA 629
```

```
Query: 544 TIY-VPHSPPTTVQATAGVRRGRAVSSTAAPLESSSDTEDTQSLPPQQLFTLLDTR--RP 600
           T+Y + H+    + A A      A +    P     +       P   L+  L TR   P
Sbjct: 630 TLYLLVHAAKQDLVAGAEATAAAAAAGAMVPQLMVGNA------PAPSLYCGLITRGNSP 683

Query: 601 ALMAAEAAATHDADSVSSASCPSDSAAIPAMDLPAGERAVLPVSPSSAADGSSANNAEMA 660
           +L A  A+     D VS+ S   +S+A  A    A        S   ++  ++A+
Sbjct: 684 SLEAGRASVPSLQD-VSARSPEIESSASSAWSSAA--------STKGSSRATNASQRRGR 734

Query: 661 TAAHTSARSDAPTENEGNELLSASSPSVAPPLSHASSSFDIGVSNGATTAVQLSSLAFQG 720
            AA  SAR+  P        L     ++    + A         N A+T  Q   +  +
Sbjct: 735 AAAERSARTRNPL---SRGYLDTEQQALVRKQALAK-------HNAASTPEQHRLVLIEA 784

Query: 721 DAVTRALLQPLIPRALDVALRVAFDYQSITLDVRYAEVRVLVYYFYSSYKILFRPLAAPE 780  Conserved
           DAVTRALLQPLIPRALDVALRVAFDYQSITLDVRYAEVRVLVYYFYSSYKILFRPLAAPE
Sbjct: 785 DAVTRALLQPLIPRALDVALRVAFDYQSITLDVRYAEVRVLVYYFYSSYKILFRPLAAPE 844

Query: 781 RHNIFRRLVTAFGVPQQGILEHLAARCVIRHVQQ 814                          region#2
           RHNIFRRLVTAFGVPQQGILEHLAAR ++  + Q
Sbjct: 845 RHNIFRRLVTAFGVPQQGILEHLAARGAVQWLSQ 878


>XP_003858709.1 hypothetical protein, conserved [Leishmania donovani]
          Length = 993

 Score =  342 bits (877), Expect = 3e-094
 Identities = 200/456 (43%), Positives = 264/456 (57%), Gaps = 26/456 (5%)

Query: 76  YRVVMVEAFAISQALEGYVMSRMLVMPNLSAPPLERIQGQNFTNFDDIGATLRRNTTMIA 135
           +R  ++ A +    +EGY+M  M  +PNL+  P +R+ GQ F  F      +  ++  I+
Sbjct: 7   FRDAILGAISAVYGVEGYIMGLMKSLPNLNETPAQRVAGQYFPKFYGYAELVSSSSPHIS 66

Query: 136 MTALQPGGVYAFFSPRNATKYGR--DTLDPN-----DNAQNFKPTPFETILSGRAVYVGP 188
           + A  PGGV     P    K+    D L+ +     D A  ++  PF TI +G     GP
Sbjct: 67  LFATAPGGVVLQVYPSEDEKFMENWDLLNSSCENHTDPAAAYREDPFTTIKNGLLALTGP 126

Query: 189 FRSTSDVLRGH----------WGLAIRRPIYNRTSYESADIRTFWGFAFTLVNISGLIDK 238
           ++S    +RG           W + +R+PIYN TS       TFWGFA    ++ GLI K
Sbjct: 127 YKSPGLPIRGWDSSSGEAHNMWWVDLRQPIYNATSTALITNSTFWGFAIVFFSVDGLIRK 186

Query: 239 N--PFAFTSLSKKRDVDYLLSVHNETSGVITVLATSLKQPTATQLADFVR-----LGTSV 291
              P    SL     + Y  S+ N + G   +LA+S+ +         F++       T
Sbjct: 187 KGLPEKMNSLEMAYII-YTASI-NGSDGCTVILASSMFKGETDCSKPFMKKFLDDATTRD 244

Query: 292 GITPNHPFLLTIRGRDYGSNLSPANIIIVVSTVLSGLLAIFAAVIAAVLWCTATYDAAAH 351
            +     + + ++     +  +P     +V T + G+  +FA  +  ++ CT  YD A H
Sbjct: 245 VLKEKLSWKIALKSMKRVNRFTPRVRNAIVITSVIGVSLLFALFMYVIVRCTRVYDGAKH 304

Query: 352 APKMAPFAMLTIGPCRGEELWDLATDQMAEVTEKLDQVLVRQMERHRAYQIQQVHPLTTS 411
           APKMAPFAMLTIGPCRGEELWDLA+DQM EVTE+L  VL RQM R+ AYQIQQVHPLTTS
Sbjct: 305 APKMAPFAMLTIGPCRGEELWDLASDQMVEVTERLGHVLARQMVRYHAYQIQQVHPLTTS 364

Query: 412 YVTRSVAAAVQMAFSTIEELQRHPIDGPLRAVLGDEVRLLLCYAVHWCTDAAVRVESLEG 471
           YVTRSVAAAVQMAFSTIEEL   PID PLR +LGDE  LLLCYAVHWCTDAAVR+E++ G
Sbjct: 365 YVTRSVAAAVQMAFSTIEELYSFPIDEPLRRLLGDEGSLLLCYAVHWCTDAAVRMEAIGG 424

Query: 472 TYRYEGCDVVFGGRMWAFAAPSVVTASEAVVQTLPC 507
             RYEG DVV+GGRMW FA P+VVT S+A + +  C
Sbjct: 425 GLRYEGPDVVYGGRMWVFAGPNVVTVSQAALPSTTC 460
```

```
 Score =  135 bits (340), Expect = 5e-032
 Identities = 113/331 (34%), Positives = 152/331 (45%), Gaps = 55/331 (16%)

Query: 551 PPTTVQATAGVRRGRAVSSTAAPLESSSDTEDTQSLPPQQLFTLLDTRRPALMAAEAAAT 610
           PP  + A A V  G + SS++A    S   E +   PP  L   L    P +    A  T
Sbjct: 648 PPRDL-AAANVVSGSSRSSSSAASVGSEKEETSPGEPPTPL---LQPVSPEVSVTAATGT 703

Query: 611 ----------HDADSVSSASCPSDSAAIPAMDLPAGERA----------VLPVSPSSAAD 650
                     H A   +++   S         +PA              ++ V P+   D
Sbjct: 704 VRPRRGQDNAHTAPGATASGSNSIGFRDSCSGIPATSSVRHHDLLLTNPLVVVPPAVTVD 763

Query: 651 ---------GSSANNAEMATAAHTSARSDAPTENEGNELLSASSPSVAPPLSHASSSFDI 701
                    G SA+   ++  +     S+ PT  + N L   ++ + A  +   S
Sbjct: 764 ATAHGLCGGGGSASTTPISGESTGGRHSNFPTCGD-NPLAHGTTAAAAAKVDALSGGITG 822

Query: 702 GVSNGATTAV-----------QLSSLAFQG-----DAVTRALLQPLIPRALDVALRVAFD 745
             SNG   AV             + A QG     D  +  LL+P I    D+ LR  FD
Sbjct: 823 LASNGGRGAVGGTPAACRPGNSEGNAALQGSGPALDNFSDLLLRPAISTQSDLLLRAVFD 882

Query: 746 YQSITLDVRYAEVRVLVYYFYSSYKILFRPLAAPERHNIFRRLVTAFGVPQQGILEHLAA 805
            Q++ LD+ Y  VRVLVYYFYSSYKILFRPLAAPE HNI+RRL+TAFGVPQQGILEHLAA
Sbjct: 883 RQAVALDLSYDSVRVLVYYFYSSYKILFRPLAAPELHNIYRRLMTAFGVPQQGILEHLAA 942

Query: 806 RCVIRHVQ--QRLKSLQFAPQH---PHLRAA 831
           RC  R +Q  +  ++L +  QH    H+R+A
Sbjct: 943 RCATRFLQRHEETQTLLWDQQHRLQMHIRSA 973
```

Supplementary Figure S4. Multiple sequence alignment in the MEGA format. Refseq IDs are shown. The list of corresponding TriTryp.DB IDs is shown in the Supplementary Table S3.

```
#mega
TITLE: adenylateCyclase

#XP_003858708_Leishmania_donovani
------------------------------------------------------------
-MMIDDAVHQRVVRPRLPPLRVQTRR------------YIGIVAFVACLLLIFVGIFSPL
IVLRNMDKSYEAEMRYIEYTLANKAVAPYRVVMVEAFAISQALEGYVMSRMLVM-PNLSA
PPLERIQGQ--NFTNFDDIGATLRRNTTMIAMTALQPGGVYAFFSPRNATKY--GRDTLD
PN---------DNAQNFKPTPFETILSGRAVYVGPFRSTSDVLR----------------
-GHWGLAIRRPIYNRTSYESA-DIRTFWGFAFTLVNISGLIDKNPFAFTSLSKKRD---V
DYLLSVH--NETSGVITVLATSL----KQPTATQLADFVRLGTSVGIT-PNHPFLLTIR-
GRDYGSNLSPANIIIVVSTVLSGLLAIFAAVIAAVLWCTATYDAAAHAPKMAPFAMLTIG
PCRGEELWDLATDQMAEVTEKLDQVLVRQMERHRAYQIQQVHPLTTSYVTRSVAAAVQMA
FSTIEELQRHPIDGPLRAVLGDEVRLLLCYAVHWCTDAAVRVESLEGTYRYEGCDVVFGG
RMWAFAAPSVVTASEAVVQTLPCFG-----------LRSIDTR-----------------
-PRCVVNVVRSRT-----------------------------------------------
------LCGATGG------------KELGGKS----------------------------
--ATIY-VPHS---PP-------------TTVQATAGVRR--------------GRAVSS
TAAPLESSSDT--------EDTQSLPPQQLFT-----------------LLD-TRRPALM
AAEAAA--------------------THDADSVSSASC----PSDSAAIPAMDLPAGERA
VL------------------------------------------------PVSPSSAADG
SSANNAEMATAAHTSARSDAPTENEGNELLSASSP----SVAPPLSHASSSFD-------
------------IGVSNG-ATTAVQLSSLAFQGDAV------------TRALLQPLIPRA
LDVALRVAFDYQSITLDVRYAEVRVLVYYFYSSYKILFRPLAAPERHNIFRRLVTAFGVP
QQGILEHLAARCVIRHVQQRLKSLQFA-PQHPHLRAAPR-----ETPLTLNAYAKLGRAT
R--------------------------------
#XP_001463486_Leishmania_infantum_JPCM5
------------------------------------------------------------
-MMIDDAVHQRVVRPRLPPLRVQTRR------------YIGIVAFVACLLLIFVGIFSPM
IVLRNMDKSYEAEMRYIEYTLANKAVAPYRVVMVEAFAISQALEGYVMSRMLVM-PNLSA
PPLERIQGQ--NFTNFDDIGATLRRNTTMIAMTALQPGGVYAFFSPRNATKY--GRDTLD
PN---------DNAQNFKPTPFETILSGRAVYVGPFRSTSDVLR----------------
-GHWGLAIRRPIYNRTSYESA-DIRTFWGFAFTLVNISGLIDKNPFAFTSLSKKRD---V
DYLLSVH--NETSGVITVLATSL----KQPTATQLAEFVRLGTSVGIT-PNHPFLLTIR-
GRDYGSNLSPANIIIVVSTVLSGLLAIFAAVIAAVLWCTATYDAAAHAPKMAPFAMLTIG
PCRGEELWDLATDQMAEVTEKLDQVLVRQMERHRAYQIQQVHPLTTSYVTRSVAAAVQMA
FSTIEELQRHPIDGPLRAVLGDEVRLLLCYAVHWCTDAAVRVESLEGTYRYEGCDVVFGG
RMWAFAAPSVVTASEAVVQTLPCFG-----------LGSIDTR-----------------
-PRCVVNVVRSRT-----------------------------------------------
------LCGATGG------------KELGGKS----------------------------
--ATIY-VPHS---PP-------------TTVQATAGVRR--------------GRAVSS
TAAPLESSSDT--------EDTQSLPPQQLFT-----------------LLD-TRRPALM
AAEAAA--------------------PHDADSVSSASC----PSDSAAIPAMDLPAGERA
V-------------------------------------------------LPVSPSSAAD
GSSANAEMATAAHTSARSDAPTENEGNELLSASSP----SVAPPLSHASSSFD-------
------------IGVSNG-ATTAVQLSSLAFQGDAV------------TRALLQPLIPRA
LDVALRVAFDYQSITLDVRYAEVRVLVYYFYSSYKILFRPLAAPERHNIFRRLVTAFGVP
QQGILEHLAARCVIRHVQQRLKSLQFA-PQHPHLRAAPR-----ETPLTLNAYAKLGRAT
R--------------------------------
#XP_001681205___Leishmania_major_strain_Friedlin
------------------------------------------------------------
-MMIDEAVYQRVVRPRLPPLRVQTRL------------YIGIVAFIACLLLIFVGIFSPL
IVLRNMDKSYEAEMRYIEYTLASKAVAPYRVVMFEAFAISQALEGYVMSRMQVL-PNLSA
PPLERIEGQ--NFTNFDDISVTLRRNMTMIAMTALQPGGVYAFFSPCNATKY--GRDTLD
PK---------DNAQNFKPTPFETILGGRAVYVGPFRYTSGVLQ----------------
-GHWGLAIRRPIYNRTSYKSA-DIHTFWGFALTLVNISGLIERNPFAFISLTTKKR--DV
```

```
DYLLSVH--NETSGAITILATSL----KQPTATQLAEFVRLGTSVGIT-PNHPFLITIR-
GRDYGSNLSPANIIIVVSTVLSGLLAIFAAVIAAVLWCTATHDTAAHAPKMAPFAMLTIG
PCRGEELWDLASDQMVEVTERLGHVLARQMVRHHAYQIQQVHPLTTSYVTRSVAAAVKMA
FSTIEELQRHPIDGPLRAVLGDEVRLLLSYAVHWCTDAAVRVESLEGTYRYEGPDVVYGG
RMWAFAAPSVVTASEAVVQTLPCFG-----------LRNIDTR-----------------
-PCCVVNVVRSRT-----------------------------------------------
------LCGATGG-----------------KS----------------------------
--ATIY-MPHS---LP-------------TTAQATAGVQC--------------GKAVSP
TAAPLESNSGT--------ENTQPLPPQQLFT-----------------LLD-TRRPALV
AAEAAA--------------------PRNVDSVSSASC----PSDSAEIPAMNLLAGECA
VL------------------------------------------------PVLPSSAADG
DSAKNAEMAIAARTSTSSDAPTESESNDILSASSL----SVAPPLSHASSSSD-------
------------IGVSNG-ATTAVRLSSLAFQGDAV------------TRALLQPLIPRA
LDVALRVAFDYQSITLDVRYAEVRVLVYYFYSSCKILFRPLAAPERHNIFRRLVTAFGVP
QQGILEHLAARCVIRYVQQRLKSRQFA-PHDPHLRVTPR-----EAPRTLNTYAKLGRAT
R--------------------------------
#XP_003872713__Leishmania_mexicana_MHOM
------------------------------------------------------------
-MMINDAVHQRVVRPRLPPLRGQTRL------------YVGIVAFVACLLLIFIGIFSPL
IVLRNMDKSYEAEMRDIEYTVANKAVAPYRVVMVEAFAISQALEGYVMSRMRVL-PNLSA
PPLERIRDQ--NFTNFDEIGITLRRNTNAIAMTALQPGGVYAFFSPSNATTY--GRDTLD
PH---------DNAQNFNPTPWETIRSGRAVYVGPFRSTSDVLR----------------
-GQWGLAVRRPIYNRTSYESA-DISTFWGFAFTLVNISGLMEENPFVFTNFTTKRR--SF
DYLLSVH--NETSDAITVLATSL----KNPTATQLAEFARLGTSVGIT-PNHPFLLTIR-
GHDYGSNLSPANIIIIVSTVLSGLFAIFAAVIAAVLWCAATYDAAAHAPKMAPFAMLTIG
PCRGEELWDLAADQMAEVTEKLDQVLGRQMERHCAYQIQQVHPLTTSYVTRSVAAAVQMA
FSTIEELQRHPIDGPLRAVLGDEGRLLLSYAVHWCTDAAVRVESLEGTYRYEGCDVVFGG
RMWALAAPNVVTASEAVVQTLPRFG-----------LCSIDTR-----------------
-SHCVVNVVRSRT-----------------------------------------------
------LCGTTGG------------KGFGGKS----------------------------
--ATIH-VPYS---PP-------------TTVQATAGARG--------------GRDISP
MATPMGGSSDI--------EDAQPLPPQQLFT-----------------LLD-TRRPALV
AAAAAA---------------------PHNANSFSASC----PSDTAVASAMDLPAGERV
IL------------------------------------------------PVSSSSTADG
GAATSPDIATAARTSASSHAPTESEGNDVFSAPSL----SVAPPLSHASSSLD-------
------------IGVPNGGATTAVRRNFLAFQGDAV------------TRALLQPLIPRA
LDVALRVAFDYQSITLDVRYAEVRVLVYYFYSSYKILFRPLAAPERHNIFRRLVTAFGVP
QQGILEHLAARCVIRYVQQRLKSRRLA-LQPPHLRASPR-----ETPLTQNVYANFGRAS
R--------------------------------
#GET86282_Leishmania_tarentolae
------------------------------------------------------------
--MMSDGVHERVARSRLLPPKAQTQL------------YIGVGAFVACLLLIFVGIFAPL
IALRNMDKNYEAMMRRTEYILASIAVAPYRMAMLEAFGISQALEGYVMSRMQVL-PNLSD
PPLDRIQGQ--NFPNFEDIGVNLRRKSSMVSMTALQPGGVYAFISPSNAAQY--GRDTLD
PN---------DNAQNFQPTPLQTIEGGRAVYVGPFLSSSQGLR----------------
-GYWGLAIRRPIFNRTDYKTA-DIHTFWGFALTLVNISGLIEKNPIGFSSFLGKKV--DV
DYLLSVS--NVTSGVITVIATSL----SHPTAAQLNEFVRLGTRVEIT-PNHPFLLTIR-
GRGYGANLSPINIMVVVSTVLSGLFILFAAFIAVVLWCMRTYDAAAHAPKLAPFAMLTIG
PCRGEELWDLAADQMAEVTEKLDQILARQMERHHAYQTQQVHPLTTSYVTRSVAAAVQMA
FGIIEELQRHPIDGPLRTVLGDDVRLQLCCAVHWCTDAVVRVESLEGTYRYEGGDVVFGG
RMWAFAAPSVVTASEAVVQTLPRFG-----------LRNIDTR-----------------
-PYCVVNVVRSRS-----------------------------------------------
------LCGATSG------------KELSSKS----------------------------
--GTIY-LPYS---PP-------------IGVTAAAGVRC--------------GRAFSW
MTAPVDGGKDA--------EDAQPLPPQQLFT-----------------LLD-MRRTALV
AAAAAA--------------------PHNVDTVLSASS----ANDSTPLPVMDMPAGDRI
VL------------------------------------------------PVSASTVTDG
VSANNAETAPVARTGTSLYPPPENEGNDLLSARSP----SVALLMSHASGSSE-------
-------------NVFSNSTIAAARRSSLAFQGDAV------------TRALLQPRIPCA
```

LDVALRVAFDYQSITLDVRYAEVRVLVYYFYSSYKILFLPLAAPERHNIFRRLVTAFGVP
QQGILEHLAARCVIRYMQQRLKSRAFA-PRSSDVRASPH-----EAPVTLNVKAKSRNAS
R--------------------------------
#XP_001562648__Leishmania_braziliensis_MHOM

-MKVTDATQPRVIRTRLPPLRAQTRL------------YIGIVAFSVCLLLIFVGVFSSL
IVLRNIDKNYEREVRLVEYTLANAAVAPFRVVMVEALGISQALEGYVMSQMTVL-PNLSA
PPAERIHGQ--NFVNFDSVGRTLVRNMTMISMTALQPGGVYAFISPANAASY--GRDTLD
IN---------DNSHNFKPTPLDTILDGHANYVGPFRSTSEILR----------------
-GYWGFAVRRPIFNRTDYSMA-DIHTFWGFTFTLVNISGILSTNPFDFSSSGVQQS--SF
DYLLSVV--NETSGVITVLETSL----QNPTAEQLEEFRKIGTTIDIA-PNLPFAVTVR-
GRDYGAHLSPTNITIVVSTALSGLLAIFATVIAAVLWCTRVYDGTKHAPKMAPFAMLTIG
PCRGEELWDLAAEQMAEVTDRLDGVLVRQMVRHGAYQIQQVHPLTTSYVTRSVAAAVQMA
FSAIEELQRRPIDGPLRAVLGDEGRLLLSYAVHWCTDAAVRVESLDGTYRYEGPDVVFGG
RMWAFAAPSVLTASEAVAQALPRFG-----------LRSVDAR-----------------
-PLRMVNVIYSWT---
------RYGTADS---------KEPNDKSAAS----------------------------
--ATIY-VPYP---LP-------------ITLQNTAGGRR--------------GSAISS
KVALIGSNSDT--------DDAQLVTQQQLFT-----------------LLD-ARRPALV
AAASVA--------------------LCNVDGFPSASH----ANKTVTVPVIDVRRSNSI
AS--SVPTFSAVDG
STTNSARFSTPTRTGASSSGPSENNGNELLSVRSL----SMGTPLQYIGGSFD-------
--------------NASNGTTKTVPVNSFAFQGDAV------------TRALLQPFITRS
LDVALRAAFDYQSITLDIRYDSVRVLVYYFYSSYKILFRPLAAPERHNIFRRLVTAFGVP
QQGILEHLAARCVIRYVQHRLKAYHST-LPHSKWPSSVC-----EAPSVLKAQ-------

#XP_010704270_Leishmania_panamensis

-MKVTDATQPRVIRTRLPPLRAQTRL------------YIGIVAFSVCLLLIFVGVFSSL
IVLRNMDKNYEREVRLVEYTLATAAVAPFRVVMVEALGISQALEGYVMSQMTVL-PNLSA
PPAERIRGQ--NFVNFDSVGRTLVRNMTMISMTALQPGGVYAFISPANAASY--GRDTLD
IN---------DNSHNFKPTPLDTILDGYANYVGPFRSTSEILR----------------
-GYWGFAVRRPIFNRTDYSMA-DIHTFWGFTFTLVNISGILSTNPFDFSASGVQQS--SF
DYLLSVV--NETSGVITVLETSL----QNPTAEQLEEFRKIGTTIDIA-PNHPFAVTVR-
GRDYGAHFSPTNITIVVSTALSGLLAIFATVIAAVLWCTRVYDGTKHAPKMAPFAMLTIG
PCRGEELWDLAAEQMAEVTDRLDGVLVRQMVRHGAYQIQQVHPLTTSYVTRSVAAAVQMA
FSAIEELQRRPIDGPLRAVLGDEGRLLLSYAVHWCTDAAVRVESLDGTYRYEGPDVVFGG
RMWAFAAPSVLTASEAVAQALPRFG-----------LRSVVAR-----------------
-PLRMVNVIYSWT---
------RYGTTDN---------KEPNDKSAAS----------------------------
--ATIY-VPYS---LP-------------ITLQNTAGGRR--------------GSAISS
KVALIGSSDT---------DDAQLVTQQQLFT-----------------LLD-TRRPALV
AAASVA--------------------LCNVDGFPSASH----ANKTVTVPVIDVRRSNNI
AS--SVPTFSAVDG
STTNSARFSTPTRTGASSSGPSENNGNELLSVRSL----SMGTPLQYIGGSFD-------
--------------NASNGTTKTAPVNSFAFQGDAV------------TRALLQPFITRS
LDVALRAAFDYQSITLDIRYDSVRVLVYYFYSSYNILFRPLAAPERHNIFRRLVTAFGVP
QQGILEHLAARCVIRYVQNRLKAYHST-LPHSKWPSSVC-----EAPSVPKAQQNHGKAF
L--------------------------------
#XP_015655949_Leptomonas_pyrrhocoris

--MLQDAKLTPSGVARARATSTQTRI------------YVLIVGFVLTLLLIFAAVFTPL
IVLKNEDAAYVSSVSRLEQQLASAAVVPFRQAIANASVITKALQGYVLSRATTL-PRLNT
SAQARMDSQRGSFNDFDAVVPTIARQVAFIAMVVLQPGGVYSMIWPAGSSLA--GRDVML
PT---------DYAHNFSPTPQDTLRGGEVAYVGPFFSSIEVMN----------------
-GIWIMAVRRPVYSRTDTKAPIDLDTFWGFTQTVVNISDVLAKNPFNFSNYINGKP-QTV
HYLLTTR--AKNTDRRVVVASSL----SDTSPEAVEEFVEHGTSVMVM-PNHYFMITVL-
GRDYGKWFSANNITIIVATATAGLFFIFAVFVALLLYCTQTYDGTVHAPKLAPFAILTVG
PCRGEELWELAPDEMADVAERLSQLLTSQMQRHHAYQIQQVHPLTTSYVTRGVAAAVQMA

```
FDAIEELHRHPIDAALQRLLGDDGCLLVSYAVHWCNDAVVRLDPMEGGYRYEGPDVVYGG
RMWAFAPPSVVTASEAVAQALPRFG-----------LCCVTAE-----------------
-PYRRVNVVRSRC-----------------------------------------------
------ACGATGD----CDMNGSSGDSSGSGT----------------------------
--ATIY-VPLS---AP-------------TTVAATAGMCRGGGG----------GGAALY
RLTAMARDTDN--------DKDTPLPPQMLLT-----------------LVD-PRRPVLM
AARAAA--------------------QAHASHRHRWPL----SMSLPNVADAEAHSPHPV
HE------------------------------------------------VVAGSGAVAR
REAGSQNPLCGVRIGGSFHS-SEEEMMEATSSAVP----GSSLP-NFGSSSSE-------
------------VGVS---GTTAVERVSLVIQGDGV------------TRALLRPVIPHA
LDVALHVAFDYQAITLGMSYNSMRVLVYYFYSSYKILFRPLAAPERHNIYRRLVTAFGVP
QQGILEHLAARCAIRCLQHSSKQRQLT-MSSRCASCNVGAV---LTPPGEHALRDSKEGL
TE-------------------------------
#XP_015655950_Leptomonas_pyrrhocoris
------------------------------------------------------------
--MMSDRKYTGLGGVHLRPHSTQVRI------------YCFIVASSACLLAVVAAICIPL
ILFQNMDNDYIANIATVEQEMAYEAAKSLSDSTMTAYSVTQALEGYVMSRMRSL-PNMSL
PPIPRVRAQ--NFSDFAYFASLLLENTENIVTAMLVPGGVRTEMAPIMQETY--MLDMFD
EK---------DSAQLFVPTPQQSVKDGKVGYIGPILYAGDYKP----------------
-RFWALAVRRPVFRYSEDNTR-SWRNFWGFTQTLLNISAVLEQRPFEMAQQKQSKKYQGL
DYVVTAL--NSRTGKLELIESSI----SHPTQDELEKFVEQGSSIAVT-PKYPFMITVR-
GRDYGKWFSANNITIIVATATAGLFFIFAVFVALLLYCTQTYDGTVHAPKLAPFAILTVG
PCRGEELWELAPDEMADVAERLSQLLTSQMQRHHAYQIQQVHPLTTSYVTRGVAAAVQMA
FDAIEELHRHPIDAALQRLLGDDGCLLVSYAVHWCNDAVVRLDPMEGGYRYEGPDVVYGG
RMWAFAPPSVVTASEAVAQALPRFG-----------LCCVTAE-----------------
-PYRRVNVVRSRC-----------------------------------------------
------ACGATGD----CDMNGSSGDSSGSGT----------------------------
--ATIY-VPLS---AP-------------TTVAATAGMCRGGGG----------GGAALY
RLTAMARDTDN--------DKDTPLPPQMLLT-----------------LVD-PRRPVLM
AARAAA--------------------QAHASHRHRWPL----SMSLPNVADAEAHSPHPV
HE------------------------------------------------VVAGSGAVAR
REAGSQNPLCGVRIGGSFHS-SEEEMMEATSSAVP----GSSLP-NFGSSSSE-------
------------VGVS---GTTAVERVSLVIQGDGV------------TRALLRPVIPHA
LDVALHVAFDYQAITLGMSYNSMRVLVYYFYSSYKILFRPLAAPERHNIYRRLVTAFGVP
QQGILEHLAARCAIRCLQHSSKQRQLV-TQRRQPETMISTVDMWETPLPGIAARPLPRKL
QKLE-----------------------------
#KPI86299_Leptomonas_seymouri
------------------------------------------------------------
--MPLYEKPVPIGSSRVRSSGVQTRI------------YVLIVAFVLTLLFIFIGIFTPL
IVFKNEDSEYTDDLAKLDQRLANDAVVPFRQAISNASMITKAMQGYIQSRMKRL-PDLNS
PAIDRINSQRGCYDDFGIVVPTIASRIDFVAKVTIQPGGVFALVWPHDESLI--GRDLFQ
PT---------DSANNFTPTPLETLHSGVVSFVGPLRLPANSME----------------
-SEWTMVVRRPIYNRTDIHAPVDIQTFWGFAASTVNISSILANNPFNFSVYFNGRK-PKV
DYLLSSK--PLKRGKRVVVASSL----PNKSPATVEAFIREGASVMVM-SNHDFLLTVRV
CRLCGTRVSANNVTVIVTAVIAGLFLIFAVFAALTIFCCRPYDAAAHAPKLAPFAMLTVG
PCRGEELWDLAPDEMVVVTDQLSELLARQMQRHHAYEIQQVHPLTTSYVTRTVSAAVQMA
FDMIEELHRRPIDDALRHLLGDDGRLLLSYAVHWCNDAVVRLDPMEGGYRYEGPDVVYGG
RMWVFAAPSVVTVSEAVEQVLPRFG-----------LGYVTVE-----------------
-SYRRVNVVRSRS-----------------------------------------------
------VCGATGD----CVINGGGSRSSSSGA----------------------------
--ATIY-VPFP---AP-------------TTVQATIGMGSLDLG----------GVAAAH
DI-----DKGD--------DREIHLPAQALMT-----------------LVD-TRRPVLV
AARDAA--------------------P--TPRQHSLPL----SASFSHVAEMGGSTPRHV
RD------------------------------------------------IVASPITASM
RDAESQNAVGNVHSGSFFHS-SEEEAVEAASSSVP----APSLP-NFGSSSSE-------
------------VGIS---GANVMERGVFVIQGDGV------------TRALLRPVIAHA
LDVALHAAFDYQSITLDVSYDSVRVLVYYFYSSYKILFRPLAAPERHNIYRRLVTAFGVP
QQGILEHLAARCAIRCLQHSAKLRQTA-TQRQRGNAGTTPV---SGPLEENTLKHSGHEA
VD-------------------------------
```

#XP_001463485__Leishmania_infantum_JPCM5
MVVTRHHAPTQQEPRASMLTGAGAYGGGGGALSGNHEGHGASAQEMVVVSPNSQEVAELP
SECASRQGHCSVVLPYRQPSPETQRFIATWQGALPKNFYIITACVLGCALTIFIGILVPM
LVIRRSANIHNAVVRAKEYDAVSAWASVLRDVMIEGFAATRALAGYSISTFPPLQTPTNA
SDSIPSDKITAYLKRFPRFASLIASKKPAVAIQAICPGGVIAMTHPHDPDTV--GRDLMS
PS---------DPTNRYKPSTRETAQSGRYAIVGPRRTTIASLK----------------
-QIWVIFTRAPLYRNTSKGVIPSQETFWGFVLLVVNVTGALDV--MDLDKLAKDHN---L
DYVLYDT--STESGDTHVIASSL---PSGAMQPDYEEFVAESTVTDVLAPHSSLHIAVR-
SRETYVSLTPTNIILIVVWTLFGSLLLLGISIAAVLWCTATYDAAAHAPKMAPFAMLTIG
PCRGEELWDLATDQMAEVTEKLDQVLVRQMERHRAYQIQQVHPLTTSYVTRSVAAAVQMA
FSTIEELQRHPIDGPLRAVLGDEVRLLLCYAVHWCTDAAVRVESLEGTYRYEGCDVVFGG
RMWAFAAPSVVTASEAVVQTLPCFG-----------LSGVVTR-----------------
-AYQTVDVMRTTR---
------ASYTDGS---EAGISDASSTIGGGGS----------------------------
--ATLYLLVHA---AK-------------PDLVAGAEATAAAAA----------AVAMVP
QLMV----------------GNAPAPSLYCGL-----------------ITR-GNSPSLE
AGRAAV--------------------P---------SL----QDVSARSPEIESSASSAW
SS--AASTKGSSRA
TNASQRRGRAAAERSARTRNPLSRGDLDTEQQALV----RKQALAKHNA-----------
-------------------ASTLEQHRLVLIEADAV------------TRALLQPLIPRA
LDVALRVAFDYQSITLDVRYAEVRVLVYYFYSSYKILFRPLAAPERHNIFRRLVTAFGVP
QQGILEHLAARGAVQWLSQVRKINGFM-HRQGSLECSSQTE---SSSVTPSPCNSLPGAQ
RLRL-----------------------------
#XP_003858707_Leishmania_donovani
MVVTRHHAPTQQEPRASMLTGAGAYGGGGGALSGNHEGHGASAQEMVVVSPNSQEVAELP
SECASRQGHCSVVLPYRQPSPETQRFIATWQGALPKNFYIITACVLGCALTIFIGILVPM
LVIRRSANIHNAVVRAKEYDAVSAWASVLRDVMIEGFAATRALAGYSISTFPPLQTPTNA
SDSIPSDKITAYLKRFPRFASLIASKKPAVAIQAICPGGVIAMTHPHDPDTV--GRDLMS
PS---------DPTNRYKPSTRETAQSGRYAIVGPRRTTIASLK----------------
-QIWVIFTRAPLYRNTSKGVIPSQETFWGFVLLVVNVTGALDV--MDLDKLAKDHN---L
DYVLYDT--STESGDTHVIASSL---PSGAMQPDYEEFVAESTVTDVLAPHSSLYIAVR-
SRETYVSLTPTNIILIVVWTLFGSLLLLGISIAAVLWCTATYDAAAHAPKMAPFAMLTIG
PCRGEELWDLATDQMAEVTEKLDQVLVRQMERHRAYQIQQVHPLTTSYVTRSVAAAVQMA
FSTIEELQRHPIDGPLRAVLGDEVRLLLCYAVHWCTDAAVRVESLEGTYRYEGCDVVFGG
RMWAFAAPSVVTASEAVVQTLPCFG-----------LSGVVTR-----------------
-AYQTVDVMRTTR---
------ASYTDGS---EAGSSDASSTIGGGGS----------------------------
--ATLYLLVHA---AK-------------QDLVAGAEATAAAAA----------AGAMVP
QLMV----------------GNAPAPSLYCGL-----------------ITR-GNSPSLE
AGRASV--------------------P---------SL----QDVSARSPEIESSASSAW
SS--AASTKGSSRA
TNASQRRGRAAAERSARTRNPLSRGYLDTEQQALV----RKQALAKHNA-----------
-------------------ASTPEQHRLVLIEADAV------------TRALLQPLIPRA
LDVALRVAFDYQSITLDVRYAEVRVLVYYFYSSYKILFRPLAAPERHNIFRRLVTAFGVP
QQGILEHLAARGAVQWLSQVRKINGFM-HRQGSLECSSQTE---SSSVTPSPCNSLPGAQ
RLRL-----------------------------
#VDZ42437_Leishmania_donovani
MVVTRHHAPTQQEPRASMLTGAGAYGGGGGALSGNHEGHGASAQEMVVVSPNSQEVAELP
SECASRQGHCSVVLPYRQPSPETQRFIATWQGALPKNFYIITACVLGCALTIFIGILVPM
LVIRRSANIHNAVVRAKEYDAVSAWASVLRDVMIEGFAATRALAGYSISTFPPLQTPTNA
SDSIPSDKITAYLKRFPRFASLIASKKPAVAIQAICPGGVIAMTHPHDPDTV--GRDLMS
PS---------DPTNRYKPSTRETAQSGRYAIVGPRRTTIASLK----------------
-QIWVIFTRAPLYRNTSKGVIPSQETFWGFVLLVVNVTGALDV--MDLDKLAKDHN---L
DYVLYDT--STESGDTHVIASSL---PSGAMQPDYEEFVAESTVTDVLAPHSSLHIAVR-
SRETYVSLTPTNIILIVLWTLFGSLLLLGISIAAVLWCTATYDAAAHAPKMAPFAMLTIG
PCRGEELWDLATDQMAEVTEKLDQVLVRQMERHRAYQIQQVHPLTTSYVTRSVAAAVQMA
FSTIEELQRHPIDGPLRAVLGDEVRLLLCYAVHWCTDAAVRVESLEGTYRYEGCDVVFGG
RMWAFAAPSVVTASEAVVQTLPCFG-----------LSGVVTR-----------------
-AYQTVDVMRTTR---

------ASYTDGS---EAGSSDASSTIGGGGS----------------------------
--ATLYLLVHA---AK-------------QDLVAGAEATAAAAA----------AGAMVP
QLMV----------------GNAPAPSLYCGL-----------------ITR-GNSPSLE
AGRASV--------------------P---------SL----QDVSARSPEIESSASSAW
SS--AASTKGSSRA
TNASQRRGRAAAERSARTRNPLSRGDLDTEQQALV----RKQALAKHNA-----------
-------------------ASTPEQHRLVLIEADAV------------TRALLQPLIPRA
LDVALRVAFDYQSITLDVRYAEVRVLVYYFYSSYKILFRPLAAPERHNIFRRLVTAFGVP
QQGILEHLAARGAVQWLSQVRKINGFM-HRQGSLECSSQTE---SSSVTPSPCNSLPGAQ
RLRL-----------------------------
#XP_003872712__Leishmania_mexicana_MHOM
MVVIRHHAPTQQEPRASMVTRAAAQGGGGGTLSSNPEGHGASAQEMVVFSPTSEGVAELA
GDCASRQGHCSVTLPYRQSPPETQRFTATWQGALPKNFYIITACILGCALTILIGILVPM
LVIRRSANLRNAVLRAKEHDAVSAWASVLRDVMIEGFAAARALAGYSISTFPPLQTPTNT
SEPMPGDQITAYLGRFPRFASLIASKKSAVALQAICPSGVIAMTYPHDPGTV--GRNLMS
PS---------DPTNRYKPSTIETAQSGRYAIVGPRRTTITSLK----------------
-RIWVIFTRAPLYRNTSQGVIPSLETFWGFVLLIVNVTGALDA--MDLGRLAKANN---L
NFVLYDT--STESGNTHVIASSL---PSATTQLDYAEFIAESTVTDILAPHSSLHIAVR-
SRETYVSLTPTNIIIIVAWTLFGSLLLLGIAIAAVLWCTATYDAAAHAPKMAPFAMLTIG
PCRGEELWDLAVDQMAEVTEKLDQVLGRQMERHCAYQIQQVHPLTTSYVTRSVAAAVQMA
FSTIEELQRHPIDGPLRAVLGDEGRLLLSYAVHWCTDAAVRVESLEGTYRYEGCDVVFGG
RMWALAAPNVVTASEAVVQTLPRFY-----------LSGVVTR-----------------
-AYQTVNVMRTTR---
------ASHTDVD---EEGVRAASNTIGGGGS----------------------------
--TTLYLLVHA---AK-------------PNLVVGAEATAAAAA----------AGAMVP
QLR----------------VGNAPASSLYCGL-----------------ITR-GNSSSLG
AGEAVS------------------------------SL----QDVSARSPEIESAAPSVG
NS--VASTKRGSRV
VNVLQCRGCAAVERTANTRHPLSREDVDTEQQVLV-----QQMLAKHNA-----------
-------------------APTPEQHRLVLIEADSV------------TRALLQPLIPRA
LDVALRVAFDYQSITLDVRYAEVRVLVYYFYSSYKILFRPLAAPERHNIFRRLVTAFGVP
QQGILEHLAARGAVQWLSQVRKINGFM-HRQGALDCSGQAE---SSSATSSPCNSLPGAQ
LLSI-----------------------------
#XP_001681204__Leishmania_major_strain_Friedlin
MVVTRHHTPTQQEPRASMLTGASAYGGGVGALSGNHEGHGASAQEMVVGSPNSQEVAELP
GECASRQGQCSLVLPYRQPSPETQKLTATWQGALPKSFYLITACVLGCALTIFIGILVPM
LVIRQSANVHSAVLRAKEHDAVSAWASVLRGVMIEGFAAARALAGYSISTFPALQTPTNA
SDSIPSDQITAYLKRFPRFASLIASRKPAVAIQAICPSGVIAMTHPHDPDSV--GRDLMS
PS---------DPTNRYKPSTTETAQSGRYAIVGPRRTTIASLK----------------
-QIWVIFTRAPLYRNTSNGVIPSLETFWGFVLLVVNVTGALDV--MDLDRLAEARN---L
DYVLYDA--STEASDTHVIASSL---PSGTMQPDYEQFIAESTVTDVLAPHSSLHIAVR-
SRETFVSLTPTNIILIVMWTLFGSLLLLGIAIAAVLWCTATHDTAAHAPKMAPFAMLTIG
PCRGEELWDLASDQMVEVTERLGHVLARQMVRHHAYQIQQVHPLTTSYVTRSVAAAVKMA
FSTIEELQRHPIDGPLRAVLGDEVRLLLSYAVHWCTDAAVRVESLEGTYRYEGPDVVYGG
RMWAFAAPSVVTASEAVVQTLPCFG-----------LSGTVTR-----------------
-AYQTVDVMRTTR---
------ASHTDGS---EAGIRNASSPIGGGGS----------------------------
--ATLYLLLHA---AK-------------PDLVAGAEATAAAAA----------PGAMAP
QLM----------------VDNAPAPSLYCGP-----------------IAS-GNSPSLE
AGAAVS------------------------------SL----QDGSARSPEIESSASSAW
SC--ATLTKGSSRA
AKASQRRERAAVERSASACNPLSRGDSHTEPQALV----QQQALAKHNA-----------
-------------------ASTPEQHSLVLIEADAV------------TRALLQPLIPRA
LDVALRVAFDYQSITLDVRYAEARVLVYYFYSSYKILFRPLAAPERHNIFRRLVTAFGVP
QQGILEHLAARGAVQWLSQVRKITGLI-HRQGSLECSSQTE---SSSVTPSPCDSLQGAQ
LLRF-----------------------------
#GET86281_Leishmania_tarentolae
-----------------MLKRACAHDSGGGTHPSHHEGHGASSQEMATLPPSSQEAAELA
GGRPARQGHCPASRTRHRFSSETQSFNASWQGALPKNFYIITACVVGCALTILIGILTPM

LVIRRSANAHDAVLREKEYDAGSSWASVLRIVMIEGFACTRALAGFAVSVFPSLQTPIDA
PDPTPIENVTEHLERFPRVASLIASKKPAVAMQYICPNGVIAATYPHDPLTV--GRNLMS
PE---------DPTNRYKPTTMEIAQSGRYAIIGPRRSTITSLD----------------
-KLWVIFTRAPLYRNTSKGMLPSLETFWGFAVLLVNVTGALDV--MDLDGLAKVKN---L
NYVLYDT--SIVTAELHVIASSL---PDGTTQEEYDKFIAESTVTDVLKPYSLLHIAVR-
SRETHVYLSPTVIISIIVWTLLGSLLLLGISIAVVLWCTRTYDAAAHAPKLAPFAMLTIG
PCRGEELWDLAADQMAEVTEKLDQILARQMERHHAYQVQQVHPLTTSYVTRSVAAAVQMA
FGIIEELQRHPIDGPLRTVLGDDVRLQLCCAVHWCTDAVVRVESLEGTYRYEGGDVVFGG
RMWAFAAPSVVTASEAVVQTLPQFG-----------LSGVGAR-----------------
-PYRTVNKKLTTR---
------ANYADDD---EGGVNDAHHTTDGSGN----------------------------
--TTLYLLVHA---AK-------------PDVVAEVEATAAASA----------AEAMVP
QPI----------------LGKDPESSLYYGM-----------------VAP-MNSPSFE
ARTAAS------------------------------SL----RDVPAQSSEAEPSAPRRW
AY--
--------AASTRANAAVDNPLSCGDLKERSA----------------------------
-------------------AVTPEQPCLVPIEVDSV------------TRALLQPRIPCA
LDVALRVAFDYQSITLDVRYAEVRVLVYYFYSSYKILFLPLAAPERHNIFRRLVTAFGVP
KQGILEHLAACSAVQWLSQVRKINRVV-YQHESL--------------------------

#GET86280_Leishmania_tarentolae
-----------------MLKRACAHDSGGGTHPSHHEGHGASSQEMATLPPSSQEAAELA
GGRPARQGHCPASRTRHRFSSETQSFNASWQGALPKNFYIITACVVGCALTILIGILTPM
LVIRRSANAHDAVLREKEYDAGSSWASVLRIVMIEGFACTRALAGFAVSVFPSLQTPIDA
PDPTPIENVTEHLERFPRVASLIASKKPAVAMQYICPNGVIAATYPHDPLTV--GRNLMS
PE---------DPTNRYKPTTMEIAQSGRYAIIGPRRSTITSLD----------------
-KLWVIFTRAPLYRNTSKGMLPSLETFWGFAVLLVNVTGALDV--MDLDGLAKVKN---L
NYVLYDT--SIVTEDQRVIASSL---PDGTTQEEYDKFIAESTVTDVLKPYSLLHIAVR-
SRETHVYLSPTVIISIIVWTLLGSLLLLGISIAVVLWCTRTYDAAAHAPKLAPFAMLTIG
PCRGEELWDLAADQMAEVTEKLDQILARQMERHHAYQTQQVHPLTTSYVTRSVAAAVQMA
FGIIEELQRHPIDGPLRTVLGDDVRLQLCCAVHWCTDAVVRVESLEGTYRYEGGDVVFGG
RMWAFAAPSVVTASEAVVQTLPQFG-----------LSGVGAR-----------------
-PYRTVNKKLTTR---
------ANYADDD---EGGVNDAHHTTDGSGN----------------------------
--TTLYLLVHA---AK-------------PDVVAEVEATAAASA----------AEAMVP
QPI----------------LGKDPESSLYYGM-----------------VAP-MNLPSFE
ARTAAS------------------------------SL----RDVPAQSSEAEPSAPRRW
AC--
--------AASTRANAAVDNPLSCGDLKERSA----------------------------
-------------------AVTPEQSCLVPIEVDSV------------TRALLQPRIPCA
LDVALRVAFDYQSITLDVRYAEVRVLVYYFYSSYKILFLPLAAPERHNIFRRLVTAFGVP
QQGILEHLAACSAVQWLSQVRKINRVV-YQHESL--------------------------

#SYZ63251_hypothetical_protein_Leishmania_braziliensis_MHOM
-------------MLERAREDNGDGGGGGSAPCGSHEAHAISTQDMVVGLPSSQRLAEPA
SGRLARQASRPVALAYHKPSPFSQKFTATPHAVLPKNFYIITACVLVCLLTIFIGILVPM
LVIRRSANARNAALRADEYSTITAWAAVLRAVMIDGLAVARALGGYALSTVPALQPSGNV
SVVLSRDNATEYLTRFPRFASLLASQKPAVALQAICPGGVIAMTYPHDAETV--GRDLMS
PS---------DPTNQHRPTTLETVLSGRYSIVGPRHTTIASLR----------------
-RIWVISTRAPLYMRTLNGTPPSLANFWGFVLLIVNVTGALDV--IQLDTLAKAKH---L
NYVIYDL--SKNSSSTRVIASSL---PPNTTQETYEAFISDSTVMDILAPLSSLYIAVR-
SRQTYVSLTATNVTIITLWTLFGSLLLLAIAIAVVLWCMRTYDAAAHAPKMAPFAMLTIG
PCRGEELWDLAAEQMAEVTDRLDGVLVRQMVRHGAYQIQQVHPLTTSYVTRSVAAAVQMA
FSAIEELQRRPIDGPLRAVLGDEGRLLLSYAVHWCTDAAVRVESLDGTYRYEGPDVVFGG
RMWAFAAPSVLTASEAVAQALPRFG-----------LSGVVAQ-----------------
-AYQMLTVVHATA---
------AKHADGNDDVEGGIRDATSTSRRSGS----------------------------
--MMLYLLAHA---AK-------------PNLLAAAAEAVAA------------AVATVP
QPM----------------IDNANSSSPSSYP-----------------IMR-MNSSSLG

```
AREAVS------------------------------GL----QDGSARPMEAFCPASTTC
NP----------------------------------------------------------
-------GCTTLRGCQRGRSPLSDGALQAEQKVVV----QSRIIVERKT-----------
-------------------DLSPEKPRVELIEADAV------------TRALLQPFITRS
LDVALRAAFDYQSITLDIRYDEVRVLVYYFYSSYSILFRPLAAPERHNIFRRLVTAFGVP
QQGILEHLAARGAMQWLSQVRKINGFM-YQQVPLECNSPTT---RTPATTSPCGTPPEAK
LMHH-----------------------------
#XP_001562647__Leishmania_braziliensis_MHOM
------------------------------------------------------------
-----------------------------------------------------------M
LVIRRSANARNAALRADEYSTITAWAAVLRAVMIDGLAVARALGGYALSTVPALQPSGNV
SVVLSRDNATEYLTRFPRFASLLASQKPAVALQAICPGGVIAMTYPHDAETV--GRDLMS
PS---------DPTNQHRPTTLETVLSGRYSIVGPRHTTIASLR----------------
-RIWVISTRAPLYMRTLNGTPPSLANFWGFVLLIVNVTGALDV--IQLDTLAKAKH---L
NYVIYDL--SKNSSSTRVIASSL---PPNTTQETYEAFISDSTVMDILAPLSSLYIAVR-
SRQTYVSLTATNVTIITLWTLFGSLLLLAIAIAVVLWCMRTYDAAAHAPKMAPFAMLTIG
PCRGEELWDLAAEQMAEVTDRLDGVLVRQMVRHGAYQIQQVHPLTTSYVTRSVAAAVQMA
FSAIEELQRRPIDGPLRAVLGDEGRLLLSYAVHWCTDAAVRVESLDGTYRYEGPDVVFGG
RMWAFAAPSVLTASEAVAQALPRFG-----------LSGVVAQ-----------------
-AYQMLTVVHATA-----------------------------------------------
------AKHADGNDDVEGGIRDATSTSRRSGS----------------------------
--MMLYLLAHA---AK-------------PNLLAAAAEAVAA------------AVATVP
QPM----------------IDNANSSSPSSYP-----------------IMR-MNSSSLG
AREAVS------------------------------GL----QDGSARPMEAFCPASTTC
NP----------------------------------------------------------
-------GCTTLRGCQRGRSPLSDGALQAEQKVVV----QSRIIVERKT-----------
-------------------DLSPEKPRVELIEADAV------------TRALLQPFITRS
LDVALRAAFDYQSITLDIRYDEVRVLVYYFYSSYSILFRPLAAPERHNIFRRLVTAFGVP
QQGILEHLAARGAMQWLSQVRKINGFM-YQQVPLECNSPTT---RTPATTSPCGTPPEAK
LMHH-----------------------------
#XP_010704269_Leishmania_panamensis
------------------------------------------------------------
-----------------------------------------------------------M
LVIRRSANARNAALRADEYSTITAWAAVLRAVMIDGLAVARALGGYALSTVPALQPSGNV
SVVLSRDNATEYLTRFPRFASLLASQKPAVALQAICPGGVIAMTYPHDAETV--GRDLMS
PN---------DPTNQYRPTTLETVLSGRYSIVGPRHTTIASLR----------------
-RIWVISTRAPLYMRTLNGTPPSLANFWGFVLLIVNVTGALDV--IQLDTLAKAKH---L
NYVIYDL--SKNSSSTRVIASSL---PPNTTQETYEAFISDSTVMDILAPLSSLYIAVR-
SRQTYVSLTATNVTIITLWTLFGSLLLLAIAIAAVLWCTRVYDGTKHAPKMAPFAMLTIG
PCRGEELWDLAAEQMAEVTDRLDGVLVRQMVRHGAYQIQQVHPLTTSYVTRSVAAAVQMA
FSAIEELQRRPIDGPLRAVLGDEGRLLLSYAVHWCTDAAVRVESLDGTYRYEGPDVVFGG
RMWAFAAPSVLTASEAVAQALPRHG-----------LSGVVTQ-----------------
-AYQMVTVVHATA-----------------------------------------------
------AKHTDGNDDVEGGIRDATSTNRRSGS----------------------------
--MMLYLLAHA---AK-------------PNLLAAAAEAVAA------------AVATVP
QLM----------------IDNANSSSPSSYP-----------------IMR-MNSSSLG
AREAVS------------------------------GL----QDGSARPMEAFCPASTTC
NL----------------------------------------------------------
-------GCTTLRGCRRGRSPLSDGALQAEQKVVV----QSRIIVECKT-----------
-------------------DLSPEQPRVELIEADAV------------TRALLQPFITRS
LDVALRAAFDYQFITLDIRYDEVRVLVYYFYSSYNILFRPLAAPERHNIFRRLVTAFGVP
QQGILEHLAARGAMQWLSQVRKINGFM-YQQVPLECNSPTT---RTPATTSPCGTPPEAK
LMHR-----------------------------
#XP_001681206__Leishmania_major_strain_Friedlin
----------------------------------------------------------MK
KIFHIPTGNSAEDEQSSKRHAKRCRV------------YFMILAVWISLLLILVNILTPM
LVLQRQDNELERMHREEEKREAMSYATTFRDAILGAISAVYGVEGYIMGLMDSL-PNLNE
TPAQRVAGQ--YFPKFYGYAQLVSSSSPHISLFATAPGGVVLQVYPSEDEDFMKNWDLLN
GSSG--NHT--DPAAAYREDPFTTIKTGLLAVTGPYKSPGLPIRGWDKSSGDAD------
```

-NMWWVDLRQPIYNATSTALI-TNSTFWGFAIVFFSVDGLVRK--KDLPEKMNSLE---M
AYIIYTASVNSSNGCTVILASSMFKGETDCSKPFMKKFLADATTRDVLKEKLSWKIALK-
SMKRVNRLTPRVRDAIVITSVIGVSLLFALFMYAIVRCTRVYDGAKHAPKMAPFAMLTIG
PCRGEELWDLASDQMVEVTERLGHVLARQMVRHHAYQIQQVHPLTTSYVTRSVAAAVQMA
FSTIEELYSFPIDEPLRRLLGDEGSLLLSYAVHWCTDAAVRMETIGGGLRYEGPDVVYGG
RMWVFAGPNVVTVSQAALPSTTCMPHVKSKLFDSVFLRGVTTRQDLYIVTDTSNHSLKEA
EAFAADQLRRSRQAQLRYVADKEADLGSTGYTRCNRLLPSYPRTEYDSSDFDSSSFAREA
SAYSEGATSGSGN-----DSNLISVVNSSGGS-------QLASGPAKRGARKDGVAGASG
V-PTAIVVPAS---TP-------------DDALVVAGASVSESASTLSRAVP--QRRMGC
RLPRDLAAANV--------VSGSPRSFSSAAS------------------VR-SEKLETS
PGEPQT--------PRLQPIAPEVSVTAATGTARPRRG----QDNAHTAPGAITSGSNSI
DFRDSCSGIH-------------------------ATSGVRRNDLLLTNPLVVVPPAVTV
A--AAAHGLCGSSGGSASTTPISGESTGGRHSNFP---TCSDNPLAY-------------
--------------------STPAACRLSNSEDNAALQGSAPALNNF-SDLLLRPAISTQ
SDLLLRTVFDRQAVALDLSYDSVRVLVYYFYSSYKILFRPLAAPELHNIYRRLMTAFGVP
QQGILEHLAARCATRFLQRHEETQTLLWDQQHRLQMHIRS----ASATAATAASISDDGV
ASTSGQAATRNTNDNKPAHTGGQEEGRKTSGGV
#XP_003872714__Leishmania_mexicana_MHOM
---M
KIFHIPTGNSAEDEQSSKRHAKRYRV------------YFTILAVWVSLLLILVTILTPM
LVLQRQDTELERMHRKEEKQQAMAYATTFRDAILGAISAVYGVEGYIMGLMQSL-PNLND
TPADRVAGQ--YFPKFYDYAELVSSSSPHISLFATAPGGVLLQVYPLEDEKFMKNWDLLN
SFSE--NHT--DPAAAYREDPFTTIKTGALALTGPYKSPGLPMRGWDSSSDEAH------
-NMWWVDLRQPIYNATSTALI-SNSTFWGFGIVFFSVDGLIRK--KGFPEAMDSLG---M
AYIIYAVSVNGSDGCTVILASSMFNGETDCSRPLMAEFLDDATTRDVLKEKLSWKIALK-
SMKRVNRFTPRVRDTIVIASVIGVLFLFVLFMYIIVRCTRVYDGAKHAPKMAPFAMLTIG
PCRGEELWDLASDQMVEVTERLGHVLARQMVRYHAYQIQQVHPLATSYVTRSVAAAVQMA
FSTIEELYNFPIDEPLRRLLGDEGRLLLSYAVHWCTDAAVRMEVMDGGFRYEGPDVVYGG
RMWVFAGPNVVTVSQAALPSTTCMPHVKSKLFDSVFLRGVTTRQDLYVVTDTSNHSLKEA
EAFAADQLRRARQAQLQYAADKEAGLGNTGYTQCNRLLTSYPRMGYDSSDLDSSSFVREA
SAYSEGAATVSGN-----DSNLISVVNSGGGQ--------VASGAAKRGARKEGVAAVGA
P-PTAIVVPAS---TP-------------DKALVVAGADGSASSRAMP------QQRMSC
RPPRDLAAANG--------VSGSSRSSSSASS-------------------A-RSEKEMS
PQEPQT--------PPLPPISSEVSVAAAAGTVRPRRG----QNNAHTAPGASKSGSNSI
GCKDNCSAI-----------P--------------AASGVRHHDLLFTNPLVVVPPAITV
A--AAAHGLCG---GSASTTPISSESTGGRHSNFP---TCGDNPLAHSTTAAA-------
-----------EKVDALPGGSTQVACMSGNSEDNAALQGGAPALDNFSSDLLLRPAISTQ
SDLLLRAVFDRQAVALDLSYDSVRVLVYYFYSSYKILFRPLAASELHNIYRRLMTAFGVP
QQGILEHLAARCATRFLQRHEETQTLLWDQQHRLQVHIRS----PSATAATATSVSDDGV
TSTSS----------------------------
#SUZ39485_Leishmania_infantum
--MK
KIFHIPTGNSAEDEQSSKRHAKRYRV------------YFTILAVWVSLLLILVTILTPI
LVLQRQDNELERMHREEEKQEAMAYATTFRDAILGAISAVYGVEGYIMGLMKSL-PNLNE
TPAQRVAGQ--YFPKFYGYAELVSSSSPHISLFATAPGGVVLQVYPSEDEKFMENWDLLN
SSCE--NHT--DPAAAYREDPFTTIKNGLLALTGPYKSPGLPIRGWDSSSGEAH------
-NMWWVDLRQPIYNATSTALI-TNSTFWGFAIVFFSVDGLIRK--KGLPEKMNSLE---M
AYIIYTASINGSDGCTVILASSMFKGETDCSKPFMKKFLDDATTRDVLKEKLSWKIALK-
SMKRVNRFTPRVRNAIVITSVIGVSLLFALFMYVIVRCTRVYDGAKHAPKMAPFAMLTIG
PCRGEELWDLASDQMVEVTERLGHVLARQMVRYHAYQIQQVHPLTTSYVTRSVAAAVQMA
FSTIEELYSFPIDEPLRRLLGDEGSLLLCYAVHWCTDAAVRMEAIGGGLRYEGPDVVYGG
RMWVFAGPNVVTVSQAALPSTTCMPHVKSKLFDSVFLRGVTTRQDLYVVTDTSNHSLKEA
EAFAADQLRRSRQAQLRYVADKEADLGSTGYTRCDRLLTSYPRTGYDSSDLDSSFFAREA
SAYSEGATSGSGN-----DSNPISVVNSSGGG-------QVASGAARRGARKDGVAGVSG
V-PTAIVVPAS---TP-------------DNALVVAGADVSESASTLSRAVP--QRRMGC
RPPRDLAAANV--------VSGSSRSSSSAAS------------------VG-SEKEETS
PGEPPT--------PLLQPVSPEVSVTAATGTVRPRRG----QDNAHTAPGATASGSNSI
GFRDSCSGIP-------------------------ATSSVRHHDLLLTNPLVVVPPAVTV
D--ATAHGLCGG-GGSASTTPISGESTGGRHSNFP---TCGDNPLAHGTTAAA---AAKV

```
DALSGGITGLASNGGRGAVGGTPAACRPGNSEGNAALQGSGPALDNF-SDLLLRPAISTQ
SDLLLRAVFDRQAVALDLSYDSVRVLVYYFYSSYKILFRPLAAPELHNIYRRLMTAFGVP
QQGILEHLAARCATRFLQRHEETQTLLWDQQHRLQMHIRS----AGATAATATSVSDDGV
TSTSG----------------------------
#AYU76476_Leishmania_donovani
----------------------------------------------------------MK
KIFHIPTGNSAEDEQSSKRHAKRYRV------------YFTILAVWVSLLLILVTILTPI
LVLQRQDNELERMHREEEKQEAMAYATTFRDAILGAISAVYGVEGYIMGLMKSL-PNLNE
TPAQRVAGQ--YFPKFYGYAELVSSSSPHISLFATAPGGVVLQVYPSEDEKFMENWDLLN
SSCE--NHT--DPAAAYREDPFTTIKNGLLALTGPYKSPGLPIRGWDSSSGEAH------
-NMWWVDLRQPIYNATSTALI-TNSTFWGFAIVFFSVDGLIRK--KGLPEKMNSLE---M
AYIIYTASINGSDGCTVILASSMFKGETDCSKPFMKKFLDDATTRDVLKEKLSWKIALK-
SMKRVNRFTPRVRNAIVITSVIGVSLLFALFMYVIVRCTRVYDGAKHAPKMAPFAMLTIG
PCRGEELWDLASDQMVEVTERLGHVLARQMVRYHAYQIQQVHPLTTSYVTRSVAAAVQMA
FSTIEELYSFPIDEPLRRLLGDEGSLLLCYAVHWCTDAAVRMEAIGGGLRYEGPDVVYGG
RMWVFAGPNVVTVSQAALPSTTCMPHVKSKLFDSVFLRGVTTRQDLYVVTDTSNHSLKEA
EAFAADQLRRSRQAQLRYVADKEADLGSTGYTRCDRLLTSYPRTGYDSSDLDSSFFAREA
SAYSEGATSGSGN-----DSNPISVVNSSGGG-------QVASGAARRGARKDGVAGVSG
V-PTAIVVPAS---TP-------------DNALVVAGADVSESASTLSRAVP--QRRMGC
RPPRDLAAANV--------VSGSSRSSSSAAS------------------VG-SEKEETS
PGEPPT--------PLLQPVSPEVSVTAATGTVRPRRG----QDNAHTAPGATASGSNSI
GFRDSCSGIP-------------------------ATSSVRHHDLLLTNPLVVVPPAVTV
D--ATAHGLCGG-GGSASTTPISGESTGGRHSNFP---TCGDNPLAHGTTAAA---AAKV
DALSGGITGLASNGGRGAVGGTPAACRPGNSEGNAALQGSGPALDNF-SDLLLRPAISTQ
SDLLLRAVFDRQAVALDLSYDSVRVLVYYFYSSYKILFRPLAAPELHNIYRRLMTAFGVP
QQGILEHLAARCATRFLQRHEETQTLLWDQQHRLQMHIRS----ASATAATATSVSDDGV
TSTSG----------------------------
#XP_001463487__Leishmania_infantum_JPCM5
------------------------------------------------------------
------------------------------------------------------------
----------------------MAYATTFRDAILGAISAVYGVEGYIMGLMKSL-PNLNE
TPAQRVAGQ--YFPKFYGYAELVSSSSPHISLFATAPGGVVLQVYPSEDEKFMENWDLLN
SSCE--NHT--DPAAAYREDPFTTIKNGLLALTGPYKSPGLPIRGWDSSSGEAH------
-NMWWVDLRQPIYNATSTALI-TNSTFWGFAIVFFSVDGLIRK--KGLPEKMNSLE---M
AYIIYTASINGSDGCTVILASSMFKGETDCSKPFMKKFLDDATTRDVLKEKLSWKIALK-
SMKRVNRFTPRVRNAIVITSVIGVSLLFALFMYVIVRCTRVYDGAKHAPKMAPFAMLTIG
PCRGEELWDLASDQMVEVTERLGHVLARQMVRYHAYQIQQVHPLTTSYVTRSVAAAVQMA
FSTIEELYSFPIDEPLRRLLGDEGSLLLCYAVHWCTDAAVRMEAIGGGLRYEGPDVVYGG
RMWVFAGPNVVTVSQAALPSTTCMPHVKSKLFDSVFLRGVTTRQDLYVVTDTSNHSLKEA
EAFAADQLRRSRQAQLRYVADKEADLGSTGYTRCDRLLTSYPRTGYDSSDLDSSFFAREA
SAYSEGATSGSGN-----DSNPISVVNSSGGG-------QVASGAARRGARKDGVAGVSG
V-PTAIVVPAS---TP-------------DNALVVAGADVSESASTLSRAVP--QRRMGC
RPPRDLAAANV--------VSGSSRSSSSAAS------------------VG-SEKEETS
PGEPPT--------PLLQPVSPEVSVTAATGTVRPRRG----QDNAHTAPGATASGSNSI
GFRDSCSGIP-------------------------ATSSVRHHDLLLTNPLVVVPPAVTV
D--ATAHGLCGG-GGSASTTPISGESTGGRHSNFP---TCGDNPLAHGTTAAA---AAKV
DALSGGITGLASNGGRGAVGGTPAACRPGNSEGNAALQGSGPALDNF-SDLLLRPAISTQ
SDLLLRAVFDRQAVALDLSYDSVRVLVYYFYSSYKILFRPLAAPELHNIYRRLMTAFGVP
QQGILEHLAARCATRFLQRHEETQTLLWDQQHRLQMHIRS----AGATAATATSVSDDGV
TSTSG----------------------------
#XP_003858709_Leishmania_donovani
------------------------------------------------------------
------------------------------------------------------------
----------------------MAYATTFRDAILGAISAVYGVEGYIMGLMKSL-PNLNE
TPAQRVAGQ--YFPKFYGYAELVSSSSPHISLFATAPGGVVLQVYPSEDEKFMENWDLLN
SSCE--NHT--DPAAAYREDPFTTIKNGLLALTGPYKSPGLPIRGWDSSSGEAH------
-NMWWVDLRQPIYNATSTALI-TNSTFWGFAIVFFSVDGLIRK--KGLPEKMNSLE---M
AYIIYTASINGSDGCTVILASSMFKGETDCSKPFMKKFLDDATTRDVLKEKLSWKIALK-
SMKRVNRFTPRVRNAIVITSVIGVSLLFALFMYVIVRCTRVYDGAKHAPKMAPFAMLTIG
```

```
PCRGEELWDLASDQMVEVTERLGHVLARQMVRYHAYQIQQVHPLTTSYVTRSVAAAVQMA
FSTIEELYSFPIDEPLRRLLGDEGSLLLCYAVHWCTDAAVRMEAIGGGLRYEGPDVVYGG
RMWVFAGPNVVTVSQAALPSTTCMPHVKSKLFDSVFLRGVTTRQDLYVVTDTSNHSLKEA
EAFAADQLRRSRQAQLRYVADKEADLGSTGYTRCDRLLTSYPRTGYDSSDLDSSFFAREA
SAYSEGATSGSGN-----DSNPISVVNSSGGG-------QVASGAARRGARKDGVAGVSG
V-PTAIVVPAS---TP-------------DNALVVAGADVSESASTLSRAVP--QRRMGC
RPPRDLAAANV--------VSGSSRSSSSAAS------------------VG-SEKEETS
PGEPPT--------PLLQPVSPEVSVTAATGTVRPRRG----QDNAHTAPGATASGSNSI
GFRDSCSGIP-------------------------ATSSVRHHDLLLTNPLVVVPPAVTV
D--ATAHGLCGG-GGSASTTPISGESTGGRHSNFP---TCGDNPLAHGTTAAA---AAKV
DALSGGITGLASNGGRGAVGGTPAACRPGNSEGNAALQGSGPALDNF-SDLLLRPAISTQ
SDLLLRAVFDRQAVALDLSYDSVRVLVYYFYSSYKILFRPLAAPELHNIYRRLMTAFGVP
QQGILEHLAARCATRFLQRHEETQTLLWDQQHRLQMHIRS----ASATAATATSVSDDGV
TSTSG----------------------------
#KPI86300_Leptomonas_seymouri
-----------------------------------------------------------M
KVFQTPTGNSAEDEQNNKRHARRYRV------------YLIILAVWIAFLVILATTLTPM
LVLQYEDTAADKAYRAEENRLANDFTTEFRDAVLNAISAVYGFQGFIMGNMKTL-PNMNG
TKEKRVEGQ--YFPDFQSYAKLVNSSTVSIAVFLTAPGGVTYQFFPKEYGAALSNWDLLE
GEQQH-PHL--NFQAMRQRSMWDAINSGKLSIAGPYRTKNLPFLEIVASKSSITLGSAMG
GETWWIDLRQPVYKATADATI-SISTFWGFAMAALDVIDLLED--NQFEETMREND---M
EYIVYTH--NKQHDCIVVVSSWP--QESDCTVPFMRDFIQTATVHDVLDDQLAWKVAVR-
SSKRGKQLTAHLRVIIVLASVLGTVGVFALVLYLIILCTRVYDGTVHAPKQAPFAMLTVG
PCRGEELWDLASDQMVAVTDRLGKVLAHQMVRHRAYQIQQVHPLTTSYVTRTVSAAVQMA
FDMIEELHRRPIDDALRHLLGDDGRLLLSYAVHWCNDAVVQLDTVEGGYRYEGPDVVYGG
RMWVFAAPSVVTVSPTALSVSQDIPHLQYKLFDSVFLRGVTERQDLYVVFDPTNQKLCEA
EALAAEQVRRARQSQRRAAESKSSDTNSN----KGHNLYSFNHNGDDGSNLDIGSFGGYP
HPHSGGSSGEGGGNRGSPDANGNAHSGSNSST---PLSGLRPRTAAAHITKKDGAVGITS
AALTPVIVPSSVMSPPLPAQRIGPAVEAASNPLVLSATDIAALP----------ERWMVP
RLGEWSDTSSNYEDAKEGDDEGKSCSFPALSDEETLYAGNKTTEAFAESQADTGKSSNTE
PQQQPTQNASLKAAQYNVCIPPPPALPPISSTARPTTSITANALADARAHGVVNHHPGSL
ADLSSRRVLDSLYGSAISSVGTSTSNTSSSLAASAIGASQRHNELSAAKSRVVVPPSAEL
TAAAAASGGHGVISKSPSCAPLPDGSITHARRITIGPTSSYDNPLAHNLATGS----AAA
AAIASAQDGAPLDVCAASASALRVAVVSFAGDGDPL-VDSRVSLDSFNSDFLLRTAITAQ
VDSLLRTVFDRQAVALDVSYDSVRVLVYYFYSSYKILFRPLAAPERHNIYRRLVTAFGVP
QQGILEHLAARCAIRFLQHHEETQTLLWDQQRRLQTHARSI---SGPSTASVSVSDDAES
SSSS-----------------------------
#SYZ63253_Leishmania_braziliensis_MHOM/BR/75/M2904
----------------------------------------------------------MK
KIYHIPTGNSAEDEQNNKRQAKRYRV------------YFIILAVLVALLLMLTTILTPM
LLLQHHDSQLEQRHRDEERLAARDYATSFRDAILGAISAVYGVEGYVMGVMKTL-PNLND
TPAQRVEGQ--YFSQFYRYAELVASAAPHISLFATAPGGVILQVYPLKEESPLKGWDLLN
SSGSGTNHT--DQAKGQRQDPYTVISTGMLDLTGPYKSSTLPALYGVNSYDEV-------
-NMWWVDLRQPIYNATSTALI-SNSTFWGFGIVVFSVDGLMQR--YNFTKVMDSNE---M
AYIVYTMDNASSGSCTVIIASPIFNGETDCNTPSMQKFIKEATTRDVLSEKISWRISLK-
STKRVNQFTPKVRNIIIISSIIGVFALFALCVCMIVRCTRVYDGTKHAPKMAPFAMLTIG
PCRGEELWDLASDQMVEVTERLGHVLAQQMVRHGAYQIQQVHPLTTSYVTRSVAAAVQMA
FSTIEELYSCPIDDPLRRLLGDEGRLLLSYAVHWCTDAAVRLEVMGSGLRYEGPDVVYSG
RMWVFAGPNVVTVSPAALPSAKRMPHVKCKLFDSVFLRGVTTRQDLYVVTDTSNHHLKEA
EAFAAEQVRRARQAQLQYTAGKETEPGSTLYTPQGRLFT-YPRIGYENSDLDNDSLCLAR
TA----ADGASAASGNRISSHPIPFANSTGGS-------HVAAGGIRKGARKDER-----
--VGSCSVPAS---TP-------------DGVLAGTGENGSESPSALSFVVP--QRRMVR
RPARKSKLANV--------VLGSPTSSSNVAN-----------------VQT-GNAVEMA
AVNPQT--------------------P---------LL----QHNATLSSSASTSGTNDI
SGNHSRSAI-----------G--------------AANNAQHPDVLPANPLVVVPPSTTM
V--AAAHGPGGGSDGSSPSTSSRNCESSVWRPGNI----PPFVGIIHSLAATQ-------
------------------------ARVRLAAEAPSA------------ARRFVPPHTTVM
AALHCRIT-----------------RLCWTTSAVTCCCAPSSPPSRTYCF----------
---------ARCLIDRLSRST---------------------------------------
```

```
---------------------------------
#XP_001562649__Leishmania_braziliensis_MHOM/BR/75/M2904
----------------------------------------------------------MK
KIYHIPTGNSAEDEQNNKRQAKRYRV------------YFIILAVLVALLLMLTTILTPM
LLLQHHDSQLEQRHRDEERLAARDYATSFRDAILGAISAVYGVEGYVMGVMKTL-PNLND
TPAQRVEGQ--YFSQFYRYAELVASAAPHISLFATAPGGVILQVYPLKEESPLKGWDLLN
SSGSGTNHT--DQAKGQRQDPYTVISTGMLDLTGPYKSSTLPALYGVNSYDEV-------
-NMWWVDLRQPIYNATSTALI-SNSTFWGFGIVVFSVDGLMQR--YNFTKVMDSNE---M
AYIVYTMDNASSGSCTVIIASPIFNGETDCNTPSMQKFIKEATTRDVLSEKISWRISLK-
STKRVNQFTPKVRNIIIISSIIGVFALFALCVCMIVRCTRVYDGTKHAPKMAPFAMLTIG
PCRGEELWDLASDQMVEVTERLGHVLAQQMVRHGAYQIQQVHPLTTSYVTRSVAAAVQMA
FSTIEELYSCPIDDPLRRLLGDEGRLLLSYAVHWCTDAAVRLEVMGSGLRYEGPDVVYSG
RMWVFAGPNVVTVSPAALPSAKRMPHVKCKLFDSVFLRGVTTRQDLYVVTDTSNHHLKEA
EAFAAEQVRRARQAQLQYTAGKETEPGSTLYTPQGRLFT-YPRIGYENSDLDNDSLCLAR
TA----ADGASAASGNRISSHPIPFANSTGGS-------HVAAGGIRKGARKDER-----
--VGSCSVPAS---TP-------------DGVLAGTGENGSESPSALSFVVP--QRRMVR
RPARKSKLANV--------VLGSPTSSSNVAN-----------------VQT-GNAVEMA
AVNPQT--------------------P---------LL----QHNATLSSSASTSGTNDI
SGNHSRSAI-----------G--------------AANNAQHPDVLPANPLVVVPPSTTM
V--AAAHGPGGGSDGSSPSTSSRNCESSVWRPGNI-PPFVGDNPLAGSNTGPG-------
-----------PTGGRGAVSSPSVCATSHNSDGSAALQDNASVLDNFSSDLLLRPVISSQ
SDLLLRAVFDRQAVALDLSYDSVRVLVYYFYSSYKILFRPLAAPELHNIYRRLVTAFGVP
QQGILEHLAARCATRYLQRHEETQTRLWDQQHRLQAHIRSL---SASTAVATSSVSDDET
ANISD----------------------------
#GET86283_Leishmania_tarentolae
----------------------------------------------------------MK
RIFHIPTGNSAEDEQTSKRHAKRYRV------------YFTILVVWVSLLLILITILTPM
LVLQRQDNEIERMHREQEKKEVMAYTTAFRDAILGAISAAYGVEGFIMGLMKAL-PNLDL
PPADRVEGQ--YFPKFYDYAELASSASPHITLFAAAPGGVILQVYPSDEEKYLSGRDLLN
IASE--SEL--DPAAAYRENPFTTIKDGSLAMTGPYKSSRLPVQGKSRIAREEQ------
-NMWWVDLRQPIYNASSTALI-SSSTFWGFAIVLFSVDGLIWD--KKFPEKMKELE---M
AYIIYTVSTNSSDDCTVILASPMFNGETDCSQPLMKSFLEDATTRDVLKEKISWRIALK-
SMKRVNRFTTRVRNAIIITSIIGVSFLFAVFMYLVLRCTRVYDGTKHAPKLAPFAMLTIG
PCRGEELWDLASDQMVEVTERLGHVLGRQMVRYHAYQIQQVHPLTTSYVTRSVAAAVQMA
FSTIEELYSFPIDEPLRRLLGDEGGLLLSYAVHWCTDAAVRVEAIGGGFRYEGPDVVYGG
RMWVFACPNVVTVSPAALPSVTCMPHVKSKLFDSVFLRGVTTRQDLYVVTDTSNHCLNEA
ETFATEQLRRAREAQLRYTADKEAELGNTGYTLCDRLLTPYPRMGYDSSDLESSSFMRDA
SAYSESATSGSGN-----DGNLMTAVNGGGGA-------QALSGASKRAIRKDGVAGVGN
V-PTAIVVPPS---TT-------------DNAQIDEGADVLELSSQLSPAVL--QLRTVC
RLPRDRAAAKL--------ASSLSKSSSNAAN-----------------V---PSEEKIS
PEAPKT--------PLLECVPSNVSVAAATGPVRQRRR----QGNAHIAPGTTTSRSNRS
GCTNNGSVIP-------------------------ATSSAPHHDLNPTSPLFVVPPAAAV
V--KSGQGMCSSSV-SSSTAPISGETIGQRQTSLT---ACSDNPLARSTIITA--ASAKT
DALAGGITSVPSNGSRGAVGSTSVACISGNFEDNAT-LQSTSVLDNFSSDLLLRPAISTQ
SDLLLRAVFDRQAVALDLSYDSVRVLVYYFYSSYKILFRPLAAPELHNIYRRLMAAFGVP
QQGILEHLAARCATRFLQRHEETQTLLFDQQHRLQMRICS----AGAAAATPASASDDGV
TNSSG----------------------------
#CCM13323_Leishmania_guyanensis
------------------------------------------------------------
---------------------------------------------------MLTTILTPM
LLLQHHDNQLEQRHRDEERLAAKDYATTFRDAILGAISAVYGVEGYIMGVMKTL-PNLND
TPAQRVEGQ--YFPQFYRYAELVASAAPHISLFATAPGGVILQVYPLKEESPLKGWDLLN
SSGSGTNHT--DPAEGQRQDPYTVISTGMLDLTGPYKSSILPTIDGEKSYDEV-------
-NMWWVDLRQPIYNATSTALI-SNSTFWGFGIVIFSVDGLMQR--YNFTKAMDINE---M
AYIVYTMDNASSGSCTVIIASPIFNGETDCNTPNMQKFIKEATTRDVLSEKISWRISLK-
STKRVNQFTPKVRNIIIISSIIGVFALFALCVCMIVRCTRVYDGTKHAPKMAPFAMLTIG
PCRGEELWDLASDQMVEVTERLGHVLAQQMVRHGAYQIQQVHPLTTSYVMRSVAAAVQMA
FSTIEELYSCPIDDPLRRLLGDEGRLLLSYAVHWCTDAAVRLEVMGSGLRYEGPDVVYSG
RMWVFAGPNVVTVSPAALPSAKRMPHVKCKLFDSVFLRGVTTRQDLYVVTDTSNHHLKEA
```

```
EAFAAEQVRRARQAQLQYTAGKETEPGSTLYTPQGRLFT-YPGIGYENSDLDNDSLCLAR
TA----ADGASAASGNRISSHPIPFANSTGGS-------HVAAGGIRKGAHKDGGV----
--------------GS-------------CSVLAGTGENGSESPSALSFVVP--QRRMVR
RPARKNKLVNV--------VLGSPTSSSNVAN-----------------VQT-GNAVEMA
AVNPQT--------------------P---------LL----QHNATLSSSASTSGTNDI
SGNHSRSAI-----------G--------------AANNAQHPDVLPTNPLVVVPPSTTM
V--AAAHGPGGGSGGSSSSTSSRNCESSVWRPGNI-PPFVGDNPLAGSNTGPG-------
-----------PTGGRGAVSSLSVCGTSHNSDGSAALQDNASVLDNFSSDLLLRPAISSQ
SDLLLRAVFDRQAVALDLSYDSVRVLVYYFYSSYKILFRPLAAPERHNIYRRLVTAFGVP
QQGILEHLAARCATRFLQRHEETQTRLWDQQHRLQAHIRSV---SASTAVATSSVSDDEA
ANISD----------------------------
#XP_010704271_Leishmania_panamensis
----------------------------------------------------------MK
KIYHIPTGNSAEDEQNNKRQAKRYRV------------YFIILAVLVALLLMLTTILTPM
LLLQHHDNQLEQRHRDEERLAAKDYATTFRDAILGAISAVYGVEGYIMGVMKTL-PNLND
TPAQRVEGQ--YFPQFYRYAELVASAAPHISLFATAPGGVILQVYPLKEESPLKGWDLLN
SSGSGTNHT--DPAEGQRQDPYTVISTGMLDLTGPYKSSILPTIDGEKSYDEV-------
-NMWWVDLRQPIYNATSTALI-SNSTFWGFGIVIFSVDGLMQR--YNFTKAMDINE---M
AYIVYTMDNASSGSCTVIIASPIFNGETDCNTPNMQKFIKEATTRDVLSEKISWRISLK-
STKRVNQFTPKVRNIIIISSIIGVFALFALCVCMIVRCTRVYDGTKHAPKMAPFAMLTIG
PCRGEELWDLASDQMVEVTERLGHVLAQQMVRHGAYQIQQVHPLTTSYVMRSVAAAVQMA
FSTIEELYSCPIDDPLRRLLGDEGRLLLSYAVHWCTDAAVRLEVMGNGLRYEGPDVVYSG
RMWVFAGPNVVTVSPAALPSAKRMPHVKCKLFDSVFLRGVTTRQDLYVVTDTSNHHLKEA
EAFAAEQVRRARQAQLQYTAGKETEPGSTLYTPQGRLFT-YPGIGYENSDLDNDSLCLAR
TA----ADGASAASGNRISSHPIPFANSTGGS-------HVAAGGIRKGARKDGG-----
--VGSCSVPAS---TP-------------DGVLAGTGENGSESPSALSFVVP--QRRMVR
RPARKNKLSNV--------VLGSPTSSSNVAN-----------------VQT-GNAVEMA
AVNPQT--------------------P---------LL----QHNATLSSSASTSGTNDI
SGNHSRSAI-----------G--------------AANNAQHPDVLPTNPLVVVPPSTTM
V--AAAHGPGGGSGGSSSSTSSRNCESSVWRPGNI-PPFVGDNPLAGSNTGPG-------
-----------PTGGRGAVSSLSVCGTSHNSDGSAALQDNASVLDNFSSDLLLRPAISSQ
SDLLLRAVFDRQAVALDLSYDSVRVLVYYFYSSYKILFRPLAAPELHNIYRRLVTAFGVP
QQGILEHLAARCATRFLQRHEETQTRLWDQQHRLQAHIRSV---SASTAVATSSVSDDEA
ANISD----------------------------
#XP_015655951_Leptomonas_pyrrhocoris
-----------------------------------------------------------M
KILQNSTGYFAEDEQNNTRHARRYRV------------YLIILAVWVALLVILAAVLTPM
LVLQHQDNNAEKASREAENELAGNLTTEFRDAVLNAISAVYGIQGFIMGEMKTL-PNITG
TKKERVTGQ--FFANFFRYANLVNSSAASMAVFLTAPGGVTYQFFPSEYGILLRNWDLLD
EDMYGPSGTWYDPAAQ-RPSPWDSIQTGRLAISGPYMTSRLPFVESKARPRNTTSGTSHP
TKTWWVDFRQPVYQPNAHATV-SISNFWGFAMAALNVDNLLED--NDFEKKMRANS---M
NYTVFTS--TKSQDCITIVSSWP--DEPDCSVPFIRNFINTASVHDVLGDQLAWKVAVK-
SAVRENRLTEHVRVAIVLSSVFGTVLIFTIMVYIIVLCTRVYDGTVHAPKGAPFAMLTVG
LCRGEELWELASDQMVEVKERLVKVLARQMQRHHAYQIQQVHPLTQSFVTHSVSAAVQMA
FDVIEELQSNPIDGVLQRLLGDDGHLLLSYAVHWCNDAVVRPEHLEGGYRYEGPDVVYGG
RMWVFAAPNVVTVSPAALHAATHIPHVQCKLFDSVFLRGVAERQDLYVLSDPTNHRLSEA
EVFAVEQVRRARQAQREIAEMKNSDADPH----KRRLTRSSNQSGYDSSDLDVGSFAG--
RPRLTGTVGGGEGNWATSSAHSNPASNSDPCSPSSPLRCRAAAAAAAKTGKREGAIGVTA
VNPTSVLVPSSSAEAA-------------TNPFVYVSDTTAAAAAAVVVLEKPQERRLVR
RPSGHGDTSSS------DEDKMSSATASTVHDDDYNELGVADKITVGLVGNR-GDRTGLP
RQKPATQTARVPTVPYATHIPVPPALPPISPAYRPVAY----TAADAAAEEEETPQQTPL
PGMRSRRALDTLY------TG--------------VGG-----EANAGKPLVVVPPSAKL
AGETAASGDRGATSRSSSSTPSSDGVVGHGRRGNVGPASSYDNPLAHNLAAGATATAAAA
AVHAAAQDGAPLDSCAPAASALRGATVPIASDGDP-LADSEVSLDTFSSDLLLRPAITVQ
ADHLLRTVFDRQAVALDVSYDSVRVLVYYFYSSYKILFRPLAAPERHNIYRRLVTAFGVP
QQGILEHLAARCAIRFLQRHDETQTLLWDQQRRLQAHARAA---SVPTATTSTSVSDDAG
GDSSN----------------------------
```
